# Color Tuning of Face-Selective Neurons in Macaque Inferior Temporal Cortex

**DOI:** 10.1523/ENEURO.0395-20.2020

**Published:** 2021-04-05

**Authors:** Marianne Duyck, Audrey L. Y. Chang, Tessa J. Gruen, Lawrence Y. Tello, Serena Eastman, Joshua Fuller-Deets, Bevil R. Conway

**Affiliations:** Laboratory of Sensorimotor Research, National Eye Institute, National Institutes of Health, Bethesda, MD 20982-4435

**Keywords:** color vision, face perception, inferior temporal cortex, inferotemporal cortex, neurophysiology, social signaling

## Abstract

What role does color play in the neural representation of complex shapes? We approached the question by measuring color responses of face-selective neurons, using fMRI-guided microelectrode recording of the middle and anterior face patches of inferior temporal cortex (IT) in rhesus macaques. Face-selective cells responded weakly to pure color (equiluminant) photographs of faces. But many of the cells nonetheless showed a bias for warm colors when assessed using images that preserved the luminance contrast relationships of the original photographs. This bias was also found for non-face-selective neurons. Fourier analysis uncovered two components: the first harmonic, accounting for most of the tuning, was biased toward reddish colors, corresponding to the L>M pole of the L-M cardinal axis. The second harmonic showed a bias for modulation between blue and yellow colors axis, corresponding to the S-cone axis. To test what role face-selective cells play in behavior, we related the information content of the neural population with the distribution of face colors. The analyses show that face-selective cells are not optimally tuned to discriminate face colors, but are consistent with the idea that face-selective cells contribute selectively to processing the green-red contrast of faces. The research supports the hypothesis that color-specific information related to the discrimination of objects, including faces, is handled by neural circuits that are independent of shape-selective cortex, as captured by the multistage parallel processing framework of IT (Lafer-Sousa and Conway, 2013).

## Significance Statement

Does the brain encode face-specific color signals, such as those related to health and emotion, through color responses of face-selective neurons? This paper addresses this question by providing the first, to our knowledge, quantitative measurements of the color-tuning of face-selective cells. Face-selective cells are not very responsive to pure color (equiluminant) pictures of faces. But both face-selective and non-face-selective cells are biased for warm colors. Information analysis shows that face-selective cells are not optimally tuned to discriminate face colors but suggests that the cells may contribute to discriminating the reddish component of faces. Alternatively, face-cell color tuning may reflect a broader adaptation of inferior temporal cortex (IT) for the detection of objects, which are, in general, characterized by warmer coloring compared with backgrounds.

## Introduction

What role does color play in the neural representation of objects? Some cells in inferior temporal cortex (IT) are shape selective, and many of these cells are also modulated by color (Komatsu and Ideura, 1993; Edwards et al., 2003). Do shape-selective IT cells play a role in discriminating among the typical colors of the shapes to which they are tuned? We take up this question by measuring the color responses of face-selective neurons in fMRI-identified face patches of macaque monkey; we evaluate the possible role of the color tuning by relating the information content at the neural population level with the distribution of face colors (Xiao et al., 2017).

The extent to which face-selective neurons in IT are color-tuned is unsettled. A widespread but not universal assumption is that face cells do not carry color information. The assumption is supported by the observation that luminance contrast is by itself sufficient for face recognition (Kemp et al., 1996; Sinha et al., 2006) and the lack of cross face/color adaptation in psychophysical experiments (Yamashita et al., 2005). Consequently, many studies of face perception use exclusively colorless images (Kanwisher et al., 1997; Ohayon et al., 2012). But while color is not essential for determining face identity, color nonetheless relays important information related to social communication, about health, emotion, and sex (Setchell and Wickings, 2005; Changizi et al., 2006; Waitt et al., 2006; Nestor and Tarr, 2008; Gerald et al., 2009; Leopold and Rhodes, 2010; Webster and MacLeod, 2011; Lefevre et al., 2013; Nakajima et al., 2017; Petersdorf et al., 2017). Moreover, faces viewed under low pressure sodium light (which impairs retinal mechanisms for encoding color) have a paradoxical appearance: they appear green, regardless of race (Hasantash et al., 2019). Such seemingly anti-Bayesian phenomena may arise if neural representations and how they are decoded are optimal with respect to the statistics of the environment (Wei and Stocker, 2015); efficient coding thus predicts a neural population whose tuning curves best discriminate among the most common face colors (Hasantash et al., 2019). One possibility is that this face-relevant color information is encoded by face-selective neurons.

To what extent could face-selective cells discriminate among face colors? Two studies have glanced the question. One found no sensitivity to color among face-selective neurons (Perrett et al., 1982). The other study found that of 22 cells, some showed higher firing rates for naturally colored face photographs compared with unnaturally colored photographs (Edwards et al., 2003), suggesting that face-selective neurons carry color signals. Quantitative measurements of color-tuning functions of face-selective neurons have, to our knowledge, not been made, which precludes an answer to the question.

**Figure 1.**
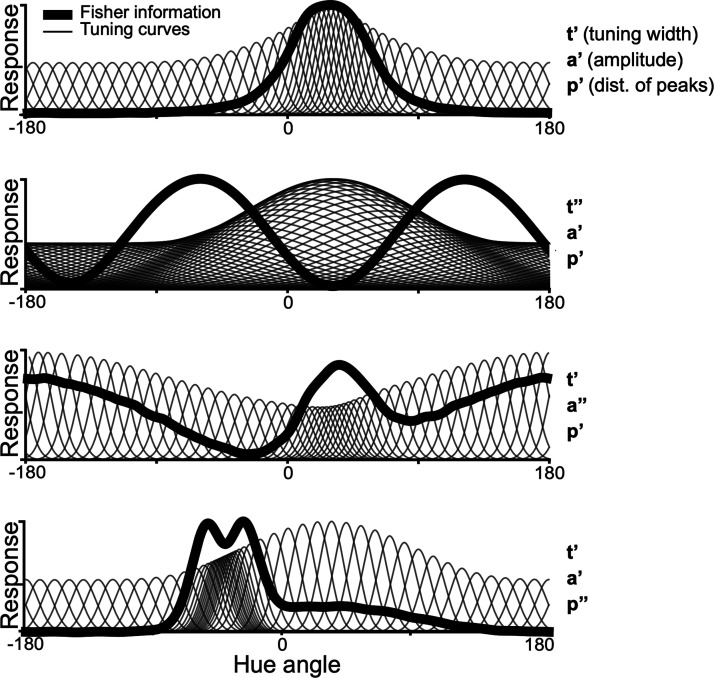
Simulated population of 50 cells responding to a circular variable and corresponding Fisher information. A population with von Mises tuning curves of width t’, amplitude a’, and distribution of peaks p’ yields a Fisher information with one peak. Each of these three parameters can be individually adjusted to create a population with a different Fisher information. Uniformly increasing tuning width (t’’) while holding a’ and p’ constant yields a two-peaked Fisher information. Independently adjusting the distribution of tuning curve amplitudes (a”) or the distribution of the peaks (p”) creates an asymmetric Fisher information.

The discrimination potential of a population can be estimated by the Fisher information, which depends on the distribution of tuning peaks, tuning widths, and amplitude modulation across the neural population (Ganguli and Simoncelli, 2010; Fig. 1). If a population activity is to be used to discriminate a given attribute, efficient coding predicts that its Fisher information should reflect the distribution of the attribute in the environment (Wei and Stocker, 2015). If face-selective cells were being used to discriminate face color, then the Fisher information across the population should reflect the natural distribution of face colors. Here, we use fMRI-guided electrode recording of neurons in the middle and anterior face patches of macaque monkeys. We discovered that face-selective cells, as a population, were biased for warm colors. The resulting Fisher Information shows a striking dip that coincides with the peak in the distribution of face colors documented in a large database of measurements of human face colors (Xiao et al., 2017). The results suggest that face-selective neurons are not optimally distributed to enable the discrimination of face colors. We discuss what role color responses of face-selective cells may play in visual processing.

## Materials and Methods

### Subjects

Three male rhesus macaques (*Macaca mulatta*), weighing 8–10 kg, were implanted with an MRI-compatible plastic (Delrin) chamber and headpost. Surgical implantation protocol has been described previously (Lafer-Sousa and Conway, 2013). Designation of the subjects are M1 (monkey 1), M2 (monkey 2), and M3 (monkey 3). M1 and M3 had chambers over the right hemisphere; M2 had a chamber on the left hemisphere. All procedures were approved by the Animal Care and Use Committee of the National Eye Institute and complied with the regulations of the National Institutes of Health.

### Functional imaging targeting of face patches

The fMRI procedures we use for localizing face patches have been described (Tsao et al., 2006; Lafer-Sousa and Conway, 2013; Rosenthal et al., 2018). Two of the animals (M1, M2) are the same as the animals used in Lafer-Sousa and Conway (2013); the face-patch data and color-tuning data are the same as in the earlier reports. Here, we present an analysis of the fMRI color-tuning data restricted to the face patches of IT. All monkeys were scanned at the Massachusetts General Hospital Martinos Imaging Center in a Siemens 3T Tim Trio scanner. Magnetic resonance images were acquired with a custom-built four-channel magnetic resonance coil system with AC88 gradient insert, which increases the signal-to-noise ratio by allowing very short echo times, providing 1-mm^3^ spatial resolution and good coverage of the temporal lobe. We used standard echo planar imaging (repetition time = 2 s, 96 × 96 × 50 matrix, 1 mm^3^ voxels; echo time = 13 ms). Monkeys were seated in a sphinx position in a custom-made chair placed inside the bore of the scanner, and they received a juice reward for maintaining fixation on a spot presented at the center of the screen at the end of the bore. An infrared eye-tracker (ISCAN) was used to monitor eye movements, and animals were only rewarded by juice for maintaining their gaze within ∼1° of the central fixation target. Magnetic resonance signal contrast was enhanced using a microparticular iron oxide agent, MION (Feraheme, 8–10 mg/kg of body weight, diluted in saline, AMAG Pharmaceuticals), injected intravenously into the femoral vein just before scanning.

Visual stimuli were displayed on a screen subtending 41 by 31 degrees of visual angle (dva), at 49 cm in front of the animal using a JVC-DLA projector (1024 × 768 pixels). The subset of presented stimuli used here to localize face patches consisted in achromatic square photographs of faces and body parts presented centrally on a neutral gray screen (∼25 cd/m^−2^) and occupying 6°. They were shown in 16 32-s blocks (16 repetition times per block, repetition time = 2 s, two images per repetition) presented in one run sequence. The images were matched in average luminance to the neutral gray, maintaining roughly constant average luminance (∼25 cd/m^−2^) throughout the stimulus sequence. For faces stimuli we used: 16 unique images front-facing of unfamiliar faces (eight human, eight monkey) repeated twice within a block. The bodies/body parts block comprised 32 unique images of monkey and human bodies (no heads/faces) and body parts. Face patches were localized by contrasting responses to faces versus responses to body parts. A total of 18 runs were used to localize face patches in M1 and M2, and 16 runs were used to localize face patches in M3.

### Physiologic recordings

A plastic grid was fitted to the inside of the recording chamber to enable us to reproducibly target regions within the brain, following details reported previously (Conway et al., 2007). We used sharp epoxy-coated tungsten electrodes (FHC), propelled using a hydraulic manual advancer (Narishige). Voltage traces were digitized and saved with a Plexon MAP system (Plexon Inc.). Spike waveforms were sorted offline with the Plexon Offline Sorter, and single units were defined on the basis of waveform and interspike interval.

Recordings were performed in a light-controlled room, with the animals seated in sphinx position. Animals were acclimatized to head restraint to minimize head movement during recordings. Animals maintained fixation on a spot on a monitor 57 cm away; the monitor was a CRT Barco subtending 40 by 30 dva, operating at 85 Hz and at a resolution of 1024 × 768 pixels. Eye position was monitored throughout the experiments using and infrared eye-tracker (ISCAN). The monitor was color calibrated using a PR-655 SpectraScan Spectroradiometer (Photograph Research Inc.); we achieved 14-bit resolution for each phosphor channel using Bits++ (Cambridge Research Systems).

### Stimuli

**Figure 2.**
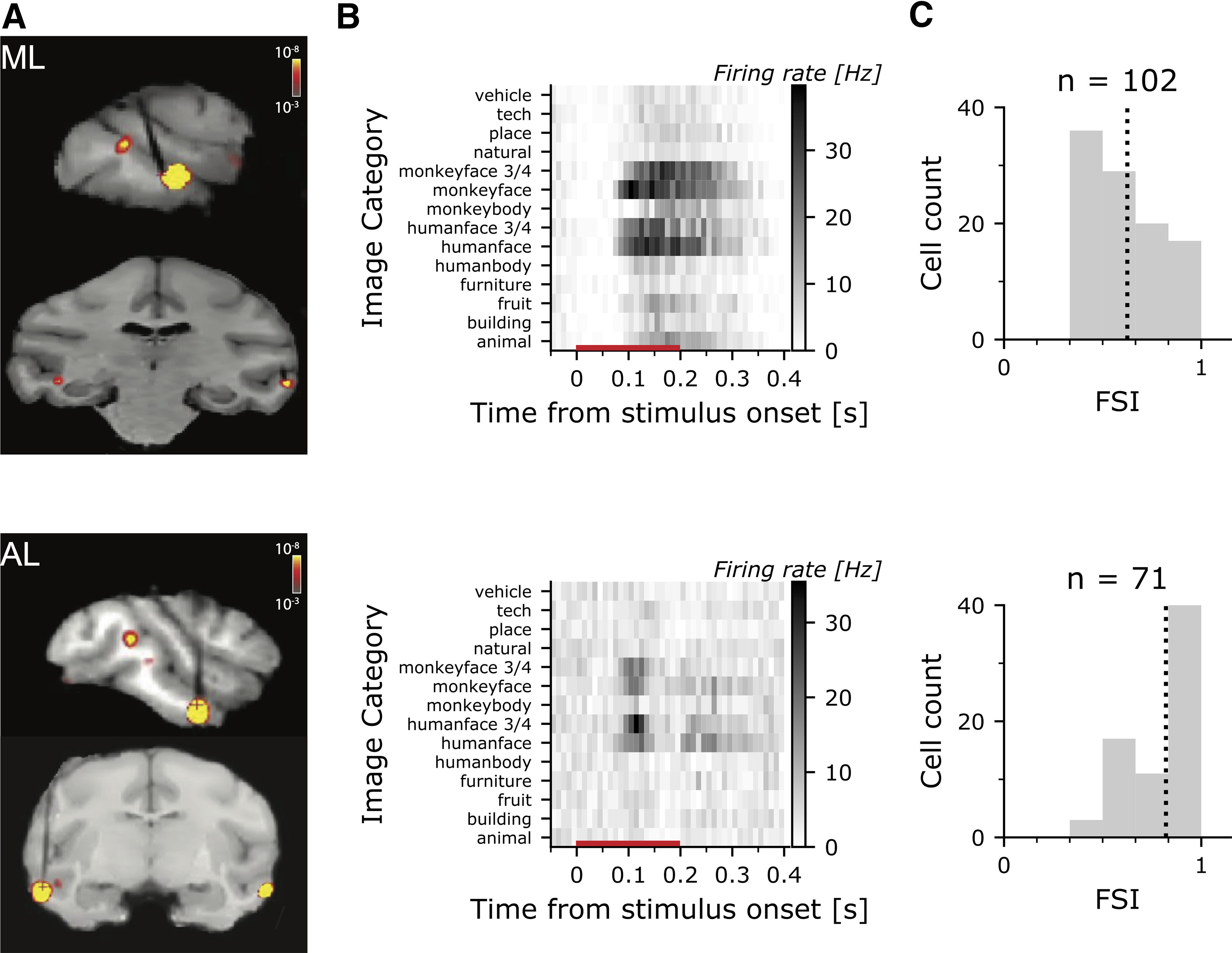
FMRI-guided microelectrode recording of face-selective cells in macaque IT. ***A***, MR Images with recording microelectrodes; superimposed is shown the fMRI contrast maps of faces>bodies uncover the face patches (ML-M2, top; AL-M3, bottom). Electrodes appear black in the MRI images, and target the face patches. ***B***, PSTH for an example cell in ML (top) and AL (bottom) showing the responses to achromatic images used to identify face cells. The red line along the *x*-axis shows the stimulus duration. ***C***, Histogram showing the FSI for the population of face cells in ML (top) and AL (bottom); the dashed vertical line shows the mean FSI. All cells with an FSI > 1/3 were included in the analysis.

Screening stimuli consisted in 10 grayscale exemplars of each of the 14 following categories: animals, buildings, human faces (front view), monkey faces (front view), human faces (3/4 view), monkey faces (3/4 view), fruits, furniture, monkey bodies (no face), human bodies (no face), places, technology objects, indoor places, natural scenes, vehicles (see examples Fig. 2). All stimuli were presented on a static luminance white noise background of 7.5 by 7.5° of visual angle.

**Figure 3.**
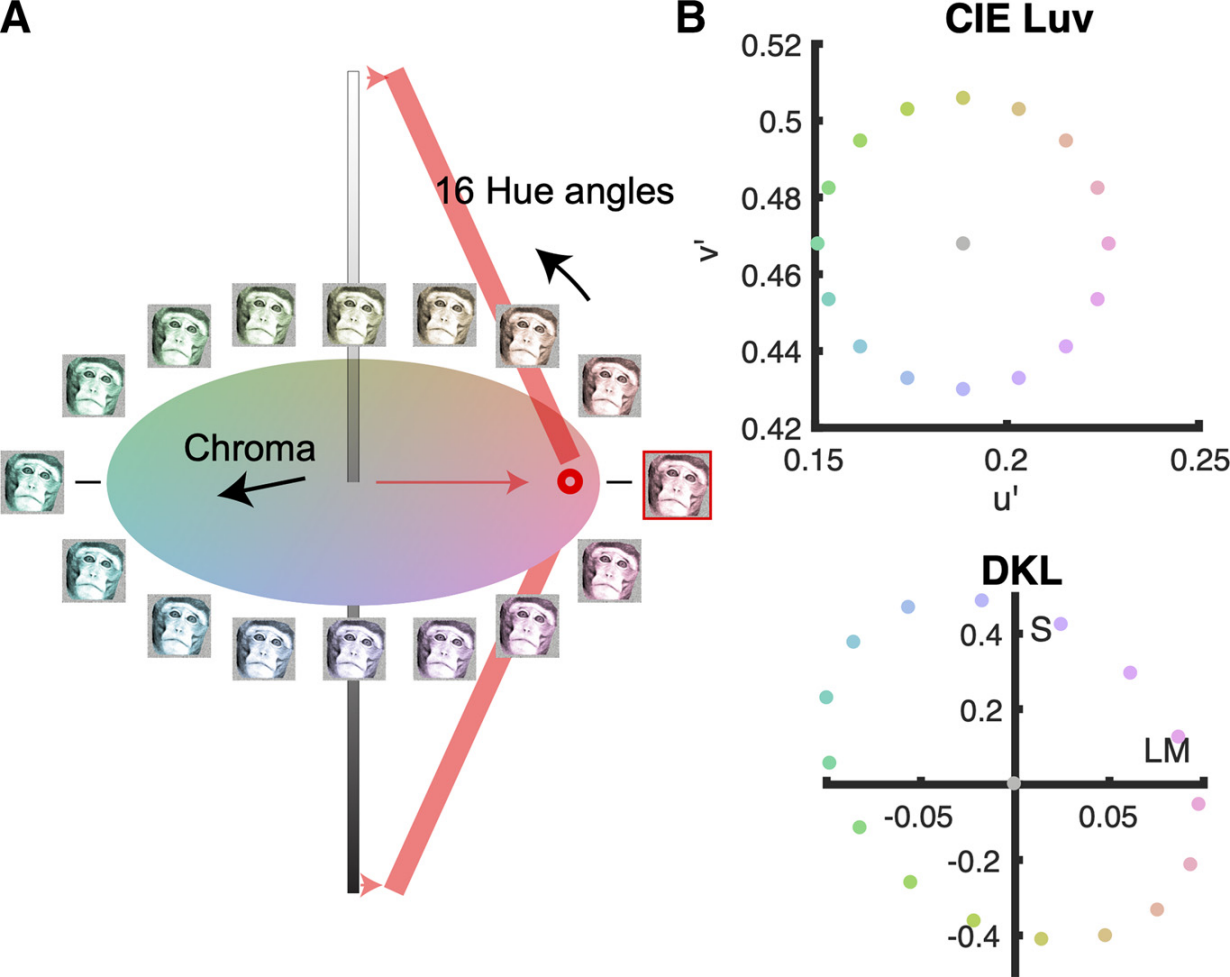
Stimuli used to measure the color responses of face-selective cells in macaque IT. ***A***, Color space illustrating the procedure for generating the colored images. Each colored image was generated by replacing the pixels in the original achromatic image with a given hue that preserved the luminance of original pixel. ***B***, Sixteen colored versions were generated; the chromaticity of the most saturated pixel in each version is shown in CIELUV (u*, v*; top panel) and cone-opponent color space (DKL, bottom panel).

Stimuli for the color-tuning experiments were exemplars of faces of unfamiliar humans and monkeys, front or 3/4 view (16 exemplar of frontal view and 8 of 3/4 view for each species), fruits known to the monkey (16 exemplars) and bodies/body parts (without head) of unfamiliar humans and monkeys (eight exemplars for each subcategory), yielding a total of 96 stimuli. For both sets of experiments using colored stimuli (main condition in which the colored stimuli preserved the luminance contrast of the original achromatic images, and the equiluminant condition), we defined 16 target hues equally spaced along the CIELUV color space (values provided in Table 1). For the main condition (Fig. 3), each pixel value of the original achromatic image was remapped to the most saturated target color of the same luminance value within the monitor gamut. For the equiluminant condition, each pixel value of the original image was remapped to a pixel on the equiluminant plane, of the same hue as the target but different saturation based on the pixel luminance. Determination of equiluminance was Judd–Vos corrected for the underestimation of the contribution of S-cones to the standard luminosity function (Vos, 1978).

**Table 1:** Coordinates of the target hues and adaptation point in CIE xyY, CIE LUV, and cone contrast spaces

	CIE xyY			CIE Luv		DKL	
	x	y	Y	u’	v’	LM	S
White point	0.3009	0.3311	56.5	0.1889	0.4677	0	0
Hue 0°	0.3473	0.3183	56.5	0.2268	0.4677	0.0979	0.0547
Hue 22.5°	0.3581	0.3427	56.5	0.2239	0.4822	0.0935	0.2145
Hue 45°	0.3607	0.3675	56.5	0.2157	0.4945	0.0760	0.3347
Hue 67.5°	0.3536	0.3884	56.5	0.2034	0.5027	0.0483	0.4026
Hue 90°	0.3370	0.4010	56.5	0.1889	0.5056	0.0145	0.4123
Hue 112.5°	0.3137	0.4020	56.5	0.1744	0.5027	–0.0214	0.3637
Hue 135°	0.2882	0.3909	56.5	0.1621	0.4945	–0.0550	0.2615
Hue 157.5°	0.2659	0.3704	56.5	0.1538	0.4822	–0.0819	0.1164
Hue 180°	0.2505	0.3450	56.5	0.1510	0.4677	–0.0979	–0.0547
Hue 202.5°	0.2441	0.3196	56.5	0.1538	0.4532	–0.0995	–0.2282
Hue 225°	0.2465	0.2980	56.5	0.1621	0.4409	–0.0852	–0.3754
Hue 247.5°	0.2563	0.2826	56.5	0.1744	0.4326	–0.0562	–0.4678
Hue 270°	0.2717	0.2747	56.5	0.1889	0.4298	–0.0171	–0.4851
Hue 292.5°	0.2907	0.2748	56.5	0.2034	0.4326	0.0248	–0.4226
Hue 315°	0.3111	0.2826	56.5	0.2157	0.4409	0.0617	–0.2933
Hue 337.5°	0.3308	0.2975	56.5	0.2239	0.4532	0.0871	–0.1239

Note that one way of making false-colored images that has been used in some studies involves the digital equivalent of superimposing a color filter over a black and white picture. In these images, the white of the original image is replaced with a relatively saturated color. Although easy to generate, these false-colored images are luminance compressed compared with the original image: the black in the colored version remains the same luminance as the original, but the white is now a lower luminance than the white in the original. Moreover, it is possible that the estimation of luminance for a given color is inaccurate (for discussion, see Conway, 2009). Such inaccuracies would introduce variability in the luminance contrast among the different colored images generated using the color-filter method. For example, when an achromatic image is falsely colored by applying a color filter, such that the white in an original image is replaced with a given equiluminant color, the resulting set of differently colored, photometrically equiluminant, versions of the image may have different luminance-contrast ranges that systematically vary by hue: the red, yellow, and green images may have a higher luminance range than the blue and purple images, because the contribution of S-cones to the luminosity function is underestimated. Thus, despite being ostensibly equiluminant, the brightest blue in the blue image may be lower luminance than the brightest red in the red image, yet the black will be the same luminance in both images. Variation in the responses to differently colored images created in this way cannot be interpreted as color tuning because they may reflect variable sensitivity to the range of luminance contrasts in the set of differently colored images. The method used presently mitigates the possible impact of chromatic aberration and variability in macular pigmentation, and gives rise to images that are more naturalistic: the images not only retain the luminance contrast of the original images but also appear differently colored, rather than achromatic but viewed through a colored filter (see Golz and MacLeod, 2002).

### Procedure

One experimental session started by targeting a microelectrode to a face patch, recording a single unit (selected online based on waveform), mapping the receptive field by hand, and choosing the visual-field location that gave the highest response to the screening stimuli. Then we recorded the neural activity while presenting the battery of screening stimuli. If a cell appeared face selective, we then recorded responses to the colored stimuli presented in random order. During both screening and main experiment stimuli were presented for 200 ms followed by a 200-ms blank (gray background). The animals were rewarded by juice for maintaining their gaze within ∼1° of the central fixation dot for a specific duration (that duration usually started at 3 s but was decreased during the experiment to adapt to the animal motivational state). Once a cell was found recording took place until the animal stopped working thus yielding a variable number of trials per session, ranging from 369 to 15,355 (Interquartile range (IQR) = [2945, 6755]). Across all sessions the number of repetitions for a given stimulus by hue combination ranged between 0 and 15. Equiluminant stimuli were presented in a subset of sessions.

### Data analysis

#### Response window

The response of each neuron was quantified within a response window defined using the average response to all stimuli, in 10-ms bins. The baseline firing rate of each cell was defined as the average response from 50 ms before stimulus onset to 10 ms after stimulus onset. The response window for each cell was determined as one continuous time period initiated when, within two consecutive time bins, the neural response increased above 2.5 SDs above the baseline firing rate and terminated either when the response dropped below 2.5 SDs of the baseline firing rate in two consecutive bins or after 200 ms following the start of the response window (the shorter of the two options was used). Cells were only included in the analysis if the response window was initiated between 50 and 250 ms after stimulus onset, and if the neural response was excitatory (i.e., cells showing suppressive responses to stimulus onset were not included).

#### Face selectivity

The present analysis focused on the color tuning of face-selective neurons. Face selectivity was assessed using the following index:

$$FSI=\frac{R_{face}-mean(R_{bodies},R_{fruits})}{\left|R_{face}|+|mean\left(R_{bodies},R_{fruits}\right)\right|},$$

where R is the average response to stimulus, computed as the difference between the firing rate during the response window and the firing rate during background. Face-selectivity index (FSI) values range from −1 to +1, with values above 0 indicating a higher response to faces compared with bodies and fruits. All analyses, with one exception, were restricted to neurons that showed an FSI ≥1/3, corresponding to a response to faces at least twice that of the response to other non-face stimuli. The exception was the last analysis (see Fig. 12), in which we examined the relationship between hue preference and face selectivity. For that last analysis we included an additional 61 cells (ML: 49 and AL: 12) that were recorded by targeting the same face patches but had an FSI below 1/3. The total number of recorded cells was thus 234 (74% of the targeted cells had an FSI of at least 1/3).

#### Color tuning

To ensure that the results are not tainted by any cells that were not face selective, we focus on the responses to color of those 173 cells that showed a FSI ≥ 1/3. An analysis of the color responses of the entire population of recorded neurons, which included some cells with low FSI, is shown in Figure 12. In the color-tuning analyses we pooled responses across the different face stimuli for each hue. Across all cells, pooling over stimuli, the number of trials per hue ranged from 10 to 634 (IQR = [119, 277]).

### Significance

We determined for each cell whether there were significant variations in net firing rate across the 16 hues by computing the coefficient of variation (the ratio of the SD across hues to the mean) of the data recorded for the neuron compared with the distribution of coefficients of variation obtained by 1000 permutations of hue labels. We considered color modulation to be significant when the *p* value was below α=0.05.

### Description: vector sum

Color responses were also quantified by determining the vector sum of the color response. This analysis is enabled because hues are circularly distributed: we can consider the neuron’s response to a hue as a vector whose direction is the hue angle, and the vector norm is the strength of the response within the response window compared with baseline. We normalized the vector norms among hues so that the total sum over hues was one. The strength of the hue preference is estimated by the direction and norm of the vector sum. Equation 1 describes the normalized vector computed for each hue; we then sum these vectors using Equation 2:

(1) $${\vec{v}}_{hue}=\frac{\left(FR_{hue}-\text{min}(FR_{hues})\right)/\left(\max\left(FR_{hues}\right)-\text{min}(FR_{hues})\right)}{\sum_{hue=1}^{16}[\left(FR_{hue}-\text{min}(FR_{hues}))/(\max\left(FR_{hues}\right)-\min\left(FR_{hues}\right)\right)]}*\left[\begin{array}{c} \cos\theta_{hue}\\ \sin\theta_{hue} \end{array}\right]$$

(2) $$\vec{v}=\overset{16}{\underset{hue=1}{\sum }}{\vec{v}}_{hue}.$$

The preferred hue direction of the cell is the angle of $\vec{v}$. The strength of the hue preference is the norm of $\vec{v},$and can take values ranging from 0 (no hue preference) to 1. The norm of the vector sum therefore reflects the narrowness of the color tuning.

### Description: Fourier analysis

Color responses can be analyzed using Fourier analysis (Krauskopf et al., 1982; Stoughton et al., 2012) that identifies the set of sine waves (frequency, phase angle, and amplitude) that capture the shape of the color-tuning function. We extracted for each cell the normalized amplitude and phase angle of the first eight harmonics; most of the power was captured by the first two harmonics. The first harmonic has a single peak when plotted in polar coordinates of color space (i.e., a vector pointing to one color), the second harmonic identifies an axis in these coordinates (i.e., the poles of the axis identify an opponent color pair). Confidence interval of the mean was obtained by resampling cells with replacement and computing the angular mean 1000 times (for the second harmonic, for all cells we used the peak between 0° and 180°).

#### Correlation between fMRI and electrophysiology

We correlated the single cell activity for each of the 16 hues for all single cells of all three monkeys separately for each face patch, to the average percent signal change to these 16 hues obtained by interpolating from the signal change to the 12 hues used in the fMRI experiment [face-patches were identified over the two hemispheres of M1 and M2 of the current study, details of stimuli and region of interest (ROI) definition can be found in Rosenthal et al., 2018]. Note that to make the Bold response and neurons’ firing rate more comparable, we averaged the net firing rate over the entire window from stimulus onset to the onset of the next stimulus (400 ms). The firing rate is thus lower than when selecting a response window tailored to each cell.

#### Population information

To compute the population of face-selective cells’ Fisher information, we fitted each cell’s net average firing rate response to the 16 hues by a von Mises function of the form r(θ)=a+b exp(κ[cos(θ−θpref)]), with κ>0, a>0 and b≥0 (median mean squared error across all cells of 0.38 spikes/s). The population Fisher information is given by:

$$I_{F}\left(\theta \right)={\left(\frac{\partial \ln\left(p\left[r\text{|}\theta \right]\right)}{\partial \theta }\right)}^{2}.$$

Assuming independent Poisson noise, we can derive that:

$$I_{F}\left(\theta \right)=\overset{173}{\underset{n=1}{\sum }}\frac{{\left(f^{\prime }{}_{n}\left(\theta \right)\right)}^{2}}{f_{n}\left(\theta \right)},$$

where f_n_ represents the tuning function for cell *n*.

For visualization, we also present the population information smoothed with a Savitsky–Golay filter (window of 50°, first order polynomial). We also computed the 95% confidence intervals of the Fisher information using non-parametric bootstrapping with 1000 iterations.

Finally, we performed the same analysis with the original 16 CIELUV hue angles projected along the two chromatic axes: 180−0° corresponding to greenish to reddish hues, and 270−90° corresponding to bluish to yellowish hues.

#### Distribution of natural face color

Xiao et al. (2017) measured the spectra of skin on the cheek, forehead, back of hand and inner arm of 960 participants of four ethnicities (White, Chinese, Kurdish, and Thai) under D65 illuminant, and reported the mean and SD of the values for each body part and each ethnicity in CIELAB color space. We averaged the forehead and cheek L, a* and b* means and SDs (Table 2 from Xiao et al., 2017 ). We then computed a weighted average across all ethnicities for both mean and SD (using Table 1 from Xiao et al., 2017). Using standard conversion matrices from CIELAB to XYZ and XYZ to CIELUV, we obtained an estimate of the mean and SD of the distribution of face color in CIELUV hue angle, represented as a von Mises distribution in Figure 11.

**Table 2:** Number of cells, median latency, and duration (in ms) of the response window used for the main analysis, by monkey and by face patch

	M1			M2			M3			Total		
	#Cells	Latency	Duration	#Cells	Latency	Duration	#Cells	Latency	Duration	#Cells	Latency	Duration
ML	45	125	180	57	75	150	-	-	-	102	95	175
AL	7	135	160	15	95	130	49	75	60	71	85	110

## Results

Functional magnetic resonance imaging was used to identify regions of IT that were more responsive to faces than to bodies and fruits, a standard contrast used to identify face patches (Tsao et al., 2006; Lafer-Sousa and Conway, 2013). We targeted microelectrodes to the ML face patch in two monkeys (M1 and M2) and the AL face patch in three monkeys (M1, M2, and M3; Fig. 2*A*). To screen for face-selective neurons, we measured the responses of each cell to a battery of grayscale images of 14 categories (Fig. 2*B*). Face-selective neurons, such as the two examples in Figure 2*B*, were defined as those that showed at least a 2-fold greater response to faces than bodies and fruits. This selection criterion yielded 102 single units in ML and 71 single units in AL. Face-selective cells in AL had a higher FSI than cells in ML (*Mdn*_ML_ = 0.60, *Mdn*_AL_ = 0.92, Mann–Whitney–Wilcoxon rank-sum test *U* = 1842, *p *<* *0.001; Fig. 2*C*); cells in AL and ML had similar firing rates (*Mdn*_ML_ = 6.38, *Mdn*_AL_ = 6.28, *U* = 3671, *p *>* *0.88). All face-selective cells showed a significant main effect of face image (repeated measures ANOVA on a cell-by-cell basis; responses were the average firing rate during the response window to each face images; analysis restricted to the 102 neurons that were tested with at least three presentations of each face image; all neurons were *p* < 0.05). The preferred face was more likely a monkey face (70% of the cells), and more likely a 3/4 view than a front view (64% of the cells). Across all cells, the preferred stimulus triggered a firing rate with a median 2.3 times higher than when using the average we are using for all analyses (IQR = [1.7, 3.0]).

We next measured responses to color for the face-selective neurons. Color responses were obtained using monochromatic versions of photographs of faces, bodies, and fruits (Fig. 3*A*); the colored stimuli evenly sampled CIELUV color space (Fig. 3*B*, top panel). We chose to define the stimuli in CIELUV color space because this space captures the representation of color within the V4 Complex (Bohon et al., 2016), which provides input into IT (Kravitz et al., 2013). The chromaticity of the stimuli can be transformed into cone-opponent “DKL” space, which reflects the cone-opponent cardinal mechanisms evident in the lateral geniculate nucleus (Derrington et al., 1984; Sun et al., 2006; Fig. 3*B*, bottom panel). Evaluating color responses in DKL space is useful because it has a physiological basis; throughout the paper, the CIE colors corresponding to the poles of the cone-opponent axes (L>M, M>L, S+, and S–) are provided to facilitate this evaluation. To create a given image in a target color, we replaced each pixel in the original gray-scale image with the target hue of the same luminance value as the pixel. Thus, the false-colored images maintained the luminance contrast of the original image.

**Figure 4.**
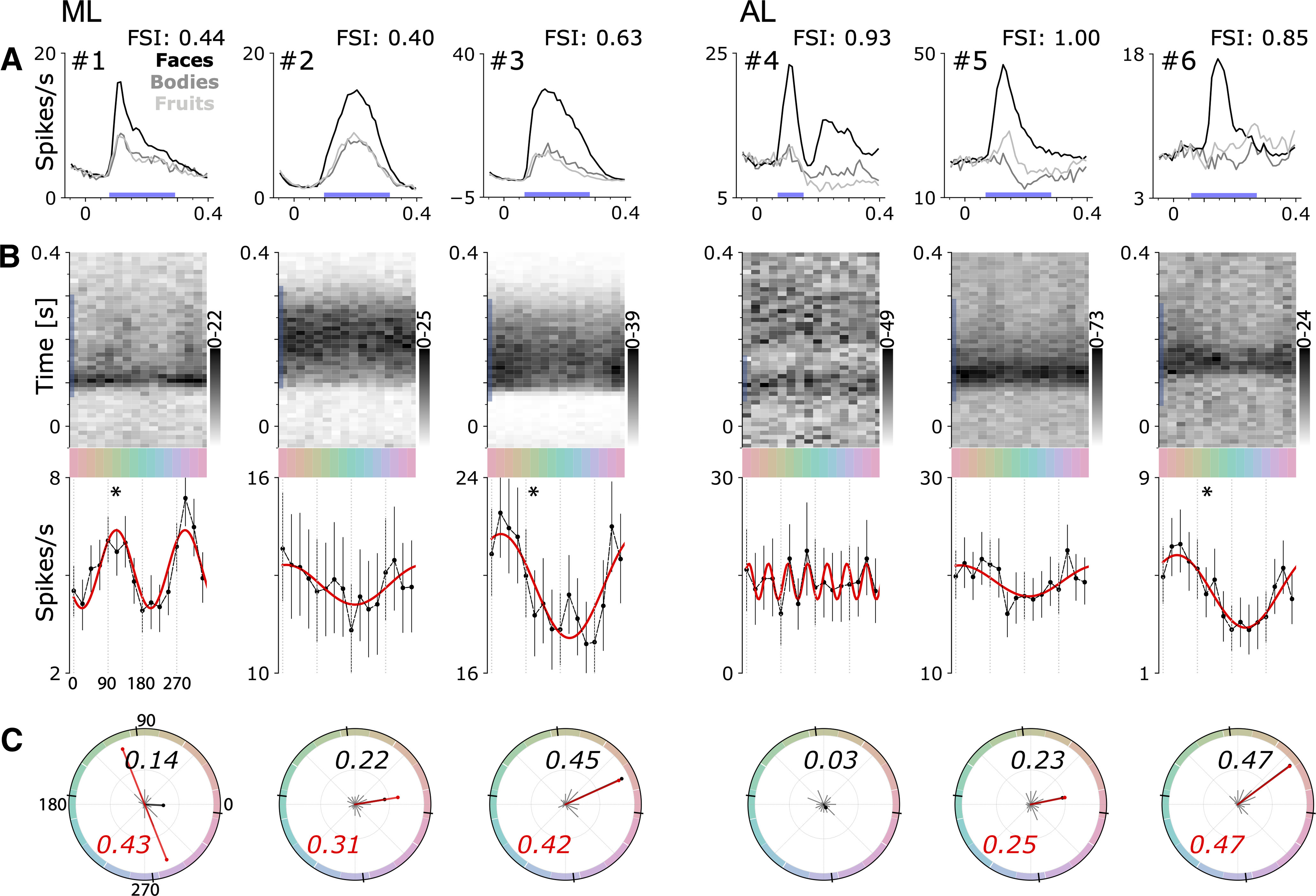
Responses to color of six face-selective cells in macaque IT (two cells for each monkey M1: #2, 3; M2: #1, 5; M3: #4, 6). ***A***, Average responses to images of faces, bodies, and fruits. The time period during which the responses were quantified in subsequent analyses is shown by the blue bar along the *x*-axis. ***B***, PSTH (top panels) showing the average responses to colored images of faces; blue bar along the time axis as in panel ***A***. Average response to face images of each color (bottom), quantified during the time period indicated by the blue bar in ***A***, ***B***. Error bars show 95% confidence intervals; the red line shows the best fitting sine wave, and an asterisk is provided if the color tuning for the neuron was significant (see Methods). ***C***, Polar plot showing normalized responses to all hues; the sum of the responses to the 16 colors is normalized to equal 1. The bold black text states the norm of the vector sum. The red line shows the normalized amplitude and phase of the best fitting sine wave for neurons whose best fit was a first or second harmonic; the red text states the value of the normalized amplitude of the best-fitting sine. The small black lines on the edges of the circle show the cardinal axes of the cone-opponent color space.

Figure 4 shows the responses of a representative sample of six face-selective neurons to the colored images of faces, bodies, and fruit; cells 1–3 were recorded in face patch ML; cells 4–6 were recorded in face patch AL. The top panels show the average responses to images of faces, bodies, and fruits. As predicted given the screening criterion, responses were always substantially larger to faces than to the other stimulus categories, with FSIs ranging from 0.44 to ∼1. For each cell, we defined a time period for quantification of the responses (Fig. 4*A*,*B*, blue bars). We used a single continuous time period for all cells whose duration was tailored to each cell. The response of cells showed complex temporal dynamics. For example, cell #4 showed two peaks (at 105 and 225 ms) and the intervening firing rate dipped back to baseline, in cells such as this one, the time period for quantification only included the initial peak. Using multiple time periods for some cells such as cell #4 did not change the main conclusions (data not shown). The median latencies and the duration of the response time periods across the population of cells are reported in Table 2. Cells in ML showed a shorter latency than cells in AL within each monkey. But the variability in latencies for cells in ML or AL across monkeys was greater than the difference in latency between ML and AL within any monkey.

Figure 4*B*, top panels, shows poststimulus time histograms (PSTHs) of the responses to the colored faces, averaging across the four different face categories we used (monkey and human faces, frontal faces and 3/4-view faces; see Fig. 2*B*). The orientation of the PSTH shows time on the *y*-axis and image color on the *x*-axis. The stimulus onset is at 0 s, and darker gray corresponds to higher firing rate. The responses of the neurons are delayed by a latency reflecting the time for visual signals to be processed by the retina and relayed through the visual-processing hierarchy to IT. The cells in Figure 4 were representative of the population: three of the cells were modulated by the color of the stimulus (cells #1, #3, #6), reflected in the average response over the response window (black trace below the PSTH; for significance calculation, see Materials and Methods). Among the population of face-selective neurons, ∼25% were significantly modulated by color (23/102 cells in ML; 21/71 cells in AL). Cells #2, #4, and #5 were not modulated significantly by color. Figure 4*B*, red traces, shows the best-fitting sine wave following Fourier analysis of the color responses, described below.

Note that the firing rates shown in Figure 4*B*, bottom panels, are averages over the response window and so are lower than the peak firing rates shown in Figure 4*A*. Although many cells were modulated by color, the variance in firing rate caused by changes in color was modest. For example, the firing rate in cell #3 varied between 18 and 22 spikes/s above background, which corresponds to ±10% of the average stimulus-driven response. Across the population, the variation in firing rate because of hue was ±24% of the average stimulus-driven response. Approximately 76% of the stimulus-driven response can be therefore attributed to the luminance contrast of the images.

**Figure 5.**
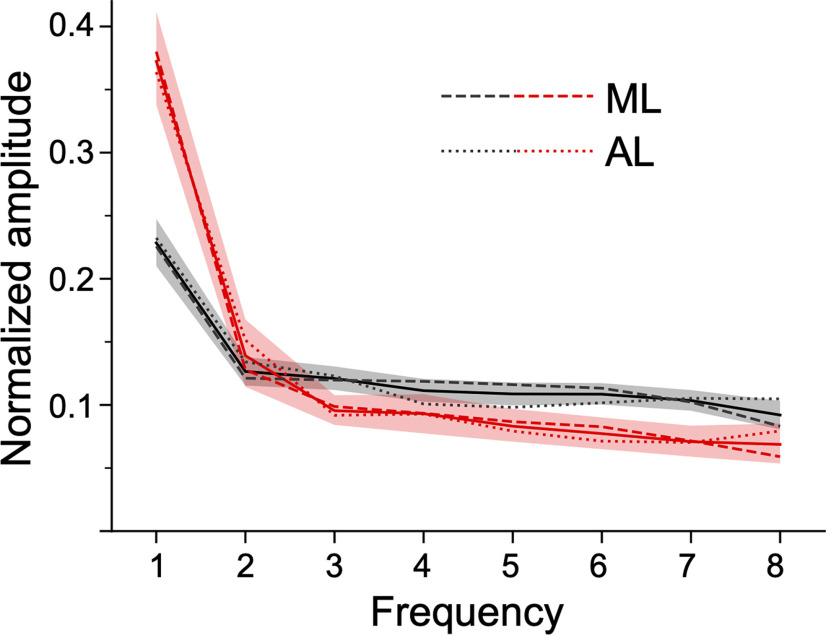
Power spectrum of the Fourier analysis of the color-tuning responses of face-selective neurons in macaque IT. Average normalized amplitude of each harmonic component for the population of 173 cells (in black) and for significantly color-tuned cells (in red). Surrounding shaded areas show 95% confidence intervals. Dashed and dotted lines represent the averages for, respectively, ML and AL and solid lines the average across both face patches.

Figure 4*C* plots the cells’ responses as a function of color angle; the norm of the vector sum is shown as the bolded black line, and varies between 0 and 1 (0 = equal net firing rate for all hues identical, i.e., no color tuning; values for each cell are shown in black text). To further quantify the results, we determined the best-fitting sine wave of the color-tuning response for each cell (Fig. 4*B*, red lines). Many cells (71/173) were best fit by the first harmonic (a single cycle), which shows that these cells have a single preferred color (see cells #3, #6; Fig. 4), but some cells were best fit by the second harmonic (21/173), indicative of a preference for a color axis in color space, rather than a single-color direction (cell #1; Fig. 4). The amplitude and phase angle of the best fitting harmonic is shown in red in Figure 4*C*. The color preferences assessed by the norm of the vector sum and the normalized amplitude of the first harmonic were highly correlated (Pearson *r* = 0.93, *p* < 0.001). Among the 44 cells showing significant color modulation, the power of both the first and the second harmonic was higher than the noise level estimated as the power to the eighth harmonic (Fig. 5, red lines); 37/44 cells showed highest power to the first harmonic; 5/44 showed highest power to the second harmonic; 1/44, to the third; and 1/44 to the fourth. We found no evidence that the color selectivity of the cells depended on the cells’ face preference: for each neuron, we computed the color selectivity (as the normalized amplitude of the first Fourier component) for each face image. We then rank-ordered the face images by descending average firing rate and ran a repeated measures ANOVA with the ranks as independent variables. There was no-significant main effect of the rank on color selectivity (*p* > 0.13).

**Figure 6.**
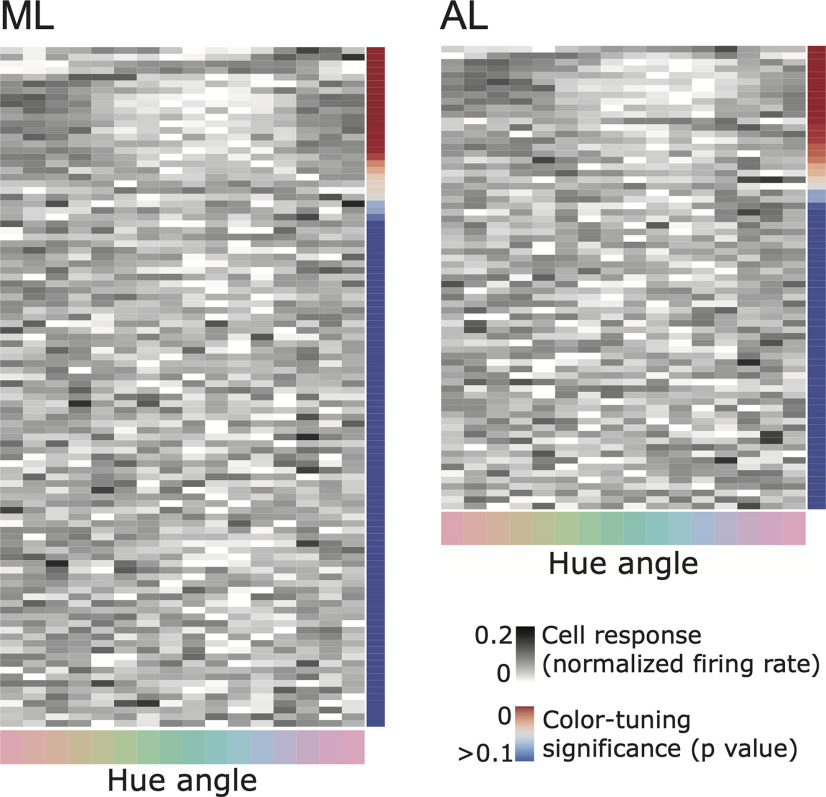
Responses to face images in each of 16 colors, for each cell in the ML face patch (left) and the AL face patch (right). Each row shows data for one cell. Cells are ordered from the top by descending color selectivity (*p* value indicated by the color scale). The plot shows normalized responses: the sum of the responses to the 16 colors for each row adds up to one (darker gray indicates relatively stronger responses).

Figure 4 provides evidence that some face-selective cells were sensitive to color. Among the population, was there a consistent color preference? Figure 6 shows the color responses of all the face-selective cells rank-ordered by the significance of the color tuning, with the most significantly color-tuned cells at the top. Each row shows data from one cell. The gray level shows the normalized response to the given color (the sum of the values across colors for a given cell is 1). Many of the most significantly color-tuned neurons in both ML and AL preferred warm colors, as evident by the dark regions on the upper left and right of the panels in Figure 6. But there were some exceptions. For example, cells represented by rows 6 and 7 in the ML panel and rows 1 and 2 in the AL panel of Figure 6 showed a preference for greenish colors.

**Figure 7.**
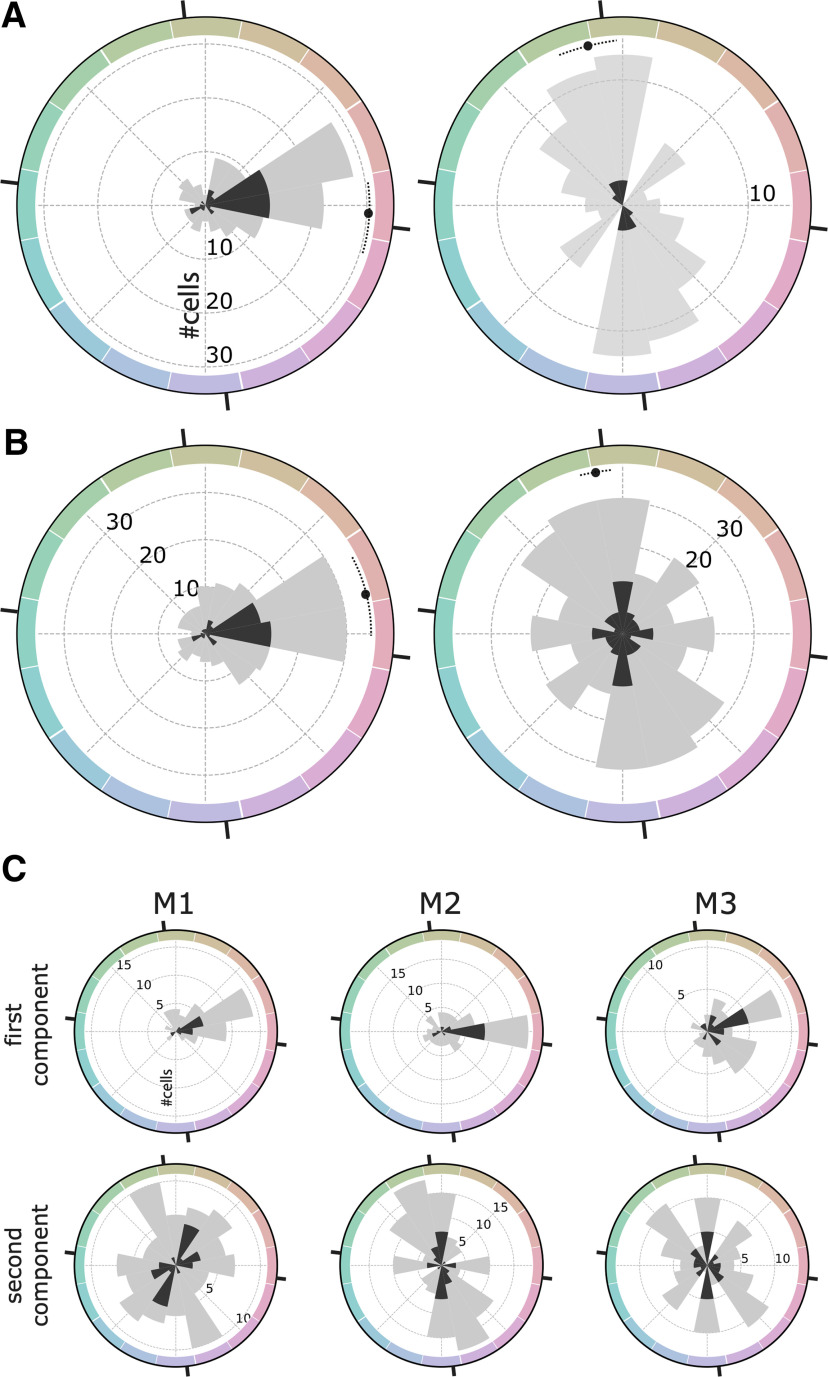
Fourier analysis of the color responses of face-selective cells in macaque IT. ***A***, left panel, Distribution of the phase angle of the first harmonic component for cells with higher amplitude in the first harmonic than the second harmonic (126/173 cells; mean: −4.40° CI = [−17.0,+8.5]). Right panel, Phase angle of the second harmonic component for cells with higher amplitude in the second harmonic compared with the first harmonic (47/173 cells; mean: +102.7° CI = [+94.5,+111.1]). ***B***, Distribution of the phase angle for the whole population (173 cells) for the first harmonic (left panel; mean +13.3° CI = [−0.1,+27.9]) and the second harmonic (right panel; mean: +99.6° CI = [+94.2,+105.3]). The color space is CIELUV; black tick marks are provided for the cardinal axes of the cone-opponent DKL color space (these are offset from the axes of CIELUV by 6.7°). ***C***, Distribution of the phase angles of the two first Fourier components across all cells for each monkey plotted separately.

Figure 7 quantifies the color responses of the population of single cells using Fourier analysis. Figure 7*A*, left panel, shows a polar histogram of the peak color direction for cells with maximum power to the first harmonic; significantly color-tuned cells are shown in dark gray. These results confirm the population bias toward the red pole of the 0−180° chromatic axis, corresponding to the L>M pole of the L-M cone-opponent axis. This bias is also evident when analyzing the color direction of the best-fitting first harmonic for all cells in the population (including those that did not have maximum power in the first harmonic; Fig. 7*B*, left panel). In contrast, cells with maximum power to the second harmonic showed a phase angle biased for the 90−270° axis, corresponding to modulation along the S-cone axis (Fig. 7*A*, right panel). And this bias was also evident when analyzing the best fitting second harmonic for all cells in the population (including those that did not have maximum power in the second harmonic; Fig. 7*B*, right panel). The pattern of results shown in Figure 7 was evident when analyzing data for each animal separately (Fig. 7*C*).

**Figure 8.**
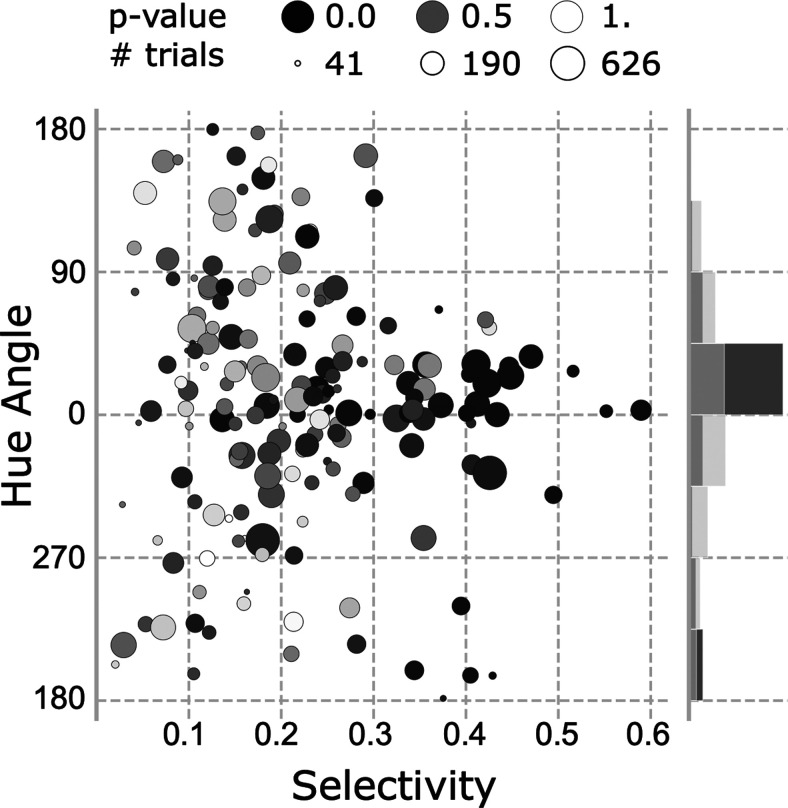
Quantification of the color responses of face-selective cells. Preferred hue angle (direction of the average vector) is plotted as a function of the strength of the color preference (norm of the average vector). Each face-selective cell is represented by a dot. The symbol size corresponds to the median number of stimulus presentations per hue. The gray value of the symbols reflects the significance of the color modulation (*p* value). The marginal distribution shows the normalized distribution of the preferred hue for significantly (dark gray) and non-significantly (light gray) color-tuned cells.

How does color tuning relate to color selectivity? If color tuning reflects a computational operation of the circuit one might predict that within the population more color-selective cells will have more consistent color tuning. Figure 8 quantifies the polar direction of the norm of the vector average (i.e., the peak color preference; *y*-axis), color selectivity (*x*-axis), significance of color tuning (symbol gray value), and number of stimulus repeats obtained (symbol size). The data points to the right of the plot converge on 0° (the L>M pole of the L-M axis), consistent with the prediction. Significantly color-tuned neurons, defined by the *p* < 0.05 threshold had a mean preferred hue angle that did not differ from insignificantly color-tuned neurons (Watson–Williams test, *F*_(1,171)_ = 0.07, *p* = 0.80) but had a significantly lower variance (marginal distribution, Wallraff test, χ^2^ = 11.59, *p* < 0.001; Fig. 8). This effect cannot be attributed to variance in the amount of data collected for different neurons. Indeed, splitting the population into two groups, above and below the median number of trials collected per cell, yielded a similar variance in the peak color for two groups (Wallraff test, χ^2^ = 1.10, *p* = 0.29).

**Figure 9.**
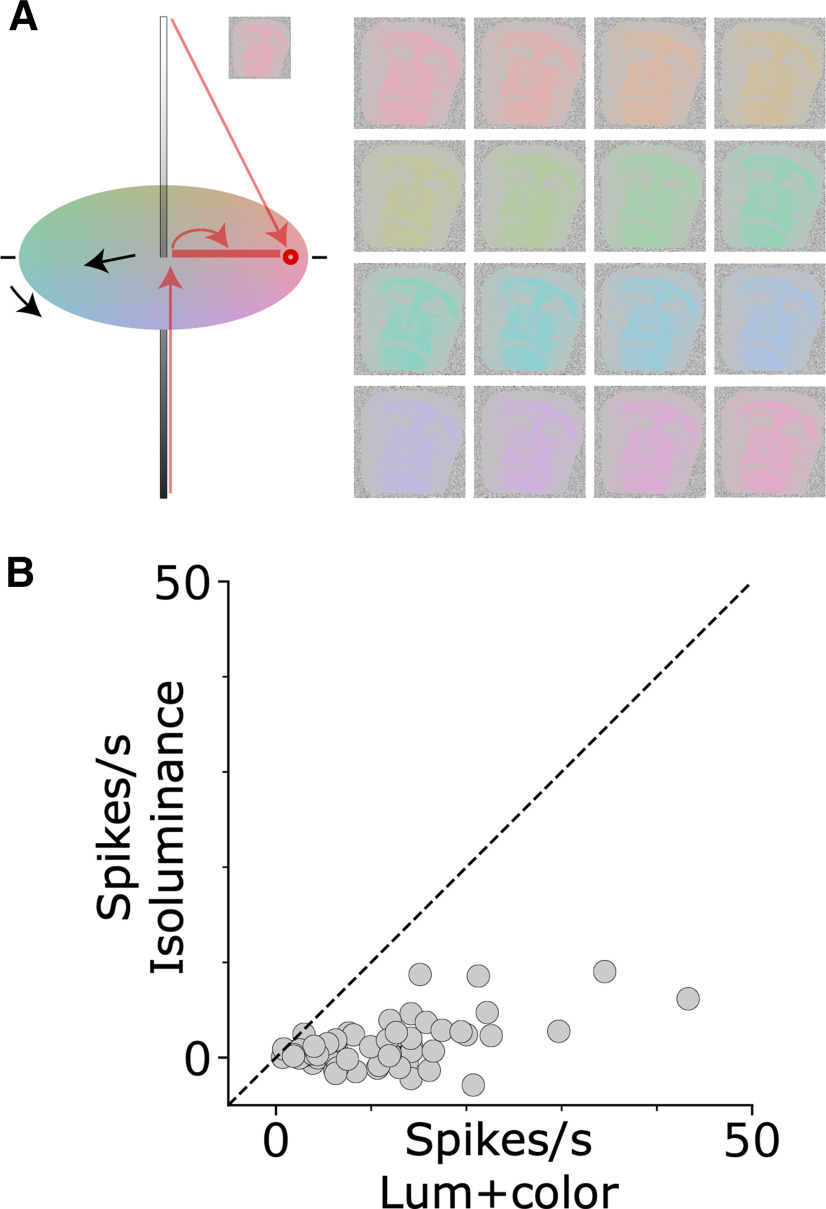
Response to equiluminant stimuli. ***A***, Illustration of the construction of an L>M colored image. The range of gray values in the original image were replaced with colors defined by a vector along an equiluminant plane in the color space; white pixels of the original image were rendered in a saturated hue, black pixels were rendered in gray, and gray pixels were rendered in a hue of intermediate saturation. ***B***, Firing rate above background of a population of face-selective neurons to equiluminant stimuli (*x*-axis) versus luminance-preserved colored stimuli (*y*-axis; *N* = 71 cells, ML = 35, AL = 36), each dot represents one cell.

The face-selective neurons responded strongly to all the colored stimuli, even those of suboptimal color (see Fig. 4). We attribute the strong color-independent responses to the luminance contrast of the stimuli (regardless of the color, the stimuli preserved the luminance contrast of the original images). We can directly dissect the role of color and luminance contrast on the cell responses by using equiluminant colored stimuli (Fig. 9*A*). These stimuli were created by replacing the range of gray values in the original images with colors of a constant gray value but different saturation: higher luminance gray values were replaced with more saturated color. Responses to these equiluminant stimuli were substantially lower than responses to the colored stimuli that preserved luminance contrast (*Mdn*_Iso_ = 0.99, *Mdn*_Main_ = 7.64, Wilcoxon rank-sum test *U* = 4643, *p* < 0.001; Fig. 9*B*). These results show that pure color is not sufficient to strongly drive face-selective cells. Because there is no accepted metric for relating color contrast and luminance contrast (Shevell and Kingdom, 2008), it is often difficult to compare responses to equiluminant stimuli with responses to luminance contrast stimuli. In the present study, this difficulty is mitigated for several reasons. First, the maximum color contrast of the equiluminant stimuli was the highest that the gamut of the display could produce. If color were a sufficient drive of the neural activity of face-selective neurons, the equiluminant stimuli we used should elicit strong responses. Second, using stimuli of comparable color and luminance contrast, other neurons in the visual system show clear preferences for the color stimuli (in V1: Conway, 2001; Johnson et al., 2004; Horwitz and Hass, 2012; in V4: Conway et al., 2007; Bohon et al., 2016; in IT: Komatsu and Ideura, 1993; Lafer-Sousa and Conway, 2013), confirming that these stimuli are capable of eliciting strong responses when neurons are responsive to color.

**Figure 10.**
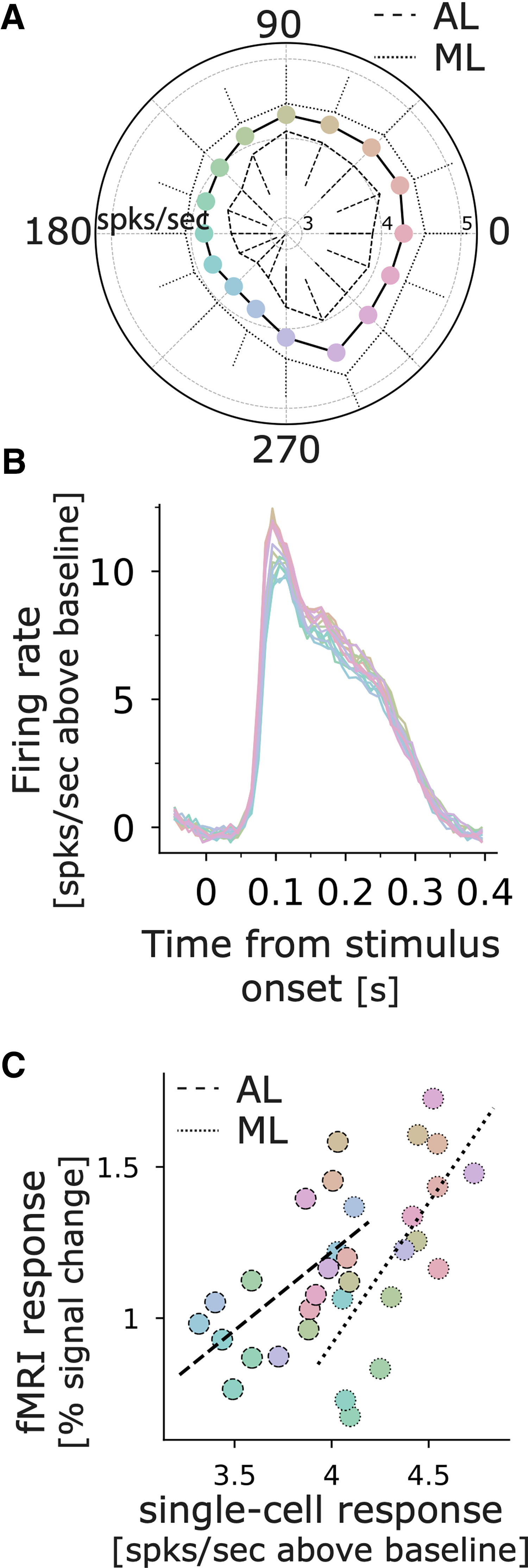
Comparison of color tuning measured using microelectrode recording of single cells in face patches and fMRI. ***A***, Average above-background firing rate computed over a 400-ms time window that begins with the stimulus onset (peak responses away from 0 can be accounted for by summing the first two harmonics of the response). ***B***, Average above-background response for all face-selective cells (*N* = 173) to face images in 16 colors (the color of the traces corresponds to the colors of the images, see Fig. 3). ***C***, Correlation between average response across the population of single units and fMRI color tuning assessed in the face patches of monkeys M1 and M2 (see Materials and Methods).

We previously measured the color responses across IT using fMRI (Lafer-Sousa and Conway, 2013; Rosenthal et al., 2018). To directly compare the results of the fMRI with the cell data, we quantified the neural responses within a 400-ms time window starting at the stimulus onset. This time window encompasses the 200-ms duration of the stimulus and the 200-ms interstimulus gray period. Figure 10*A* shows the average response for the population, in ML (solid line) and AL (dotted line). The plot shows significant differences among the responses to the colors (non-parametric Friedman test, χ^2^ = 210.69, *p* < 0.001) and the responses in ML are highly correlated with those in AL (Pearson *r* = 0.89, *p* < 0.001). Figure 10*B* shows the average response across all face-selective cells to face images in each of the 16 colors. This plot underscores two main conclusions. First, responses to all colored images were strong, which we attribute to the fact that all the colored exemplars preserved the luminance contrast of the original achromatic images, the luminance contrast is a main determinant of face-selective responses (Ohayon et al., 2012). And second, among the colored stimuli, responses to the L>M stimuli (appearing pinkish) were higher than response to the M<L stimuli (appearing greenish). The color biases of the population of face-selective cells were strongly correlated with the color biases of the face patches measured with fMRI (ML: Pearson *r* = 0.67, *p* = 0.005, power = 0.84; AL: Pearson *r* = 0.61 *p* = 0.01, power = 0.75; Fig. 10*C*).

**Figure 11.**
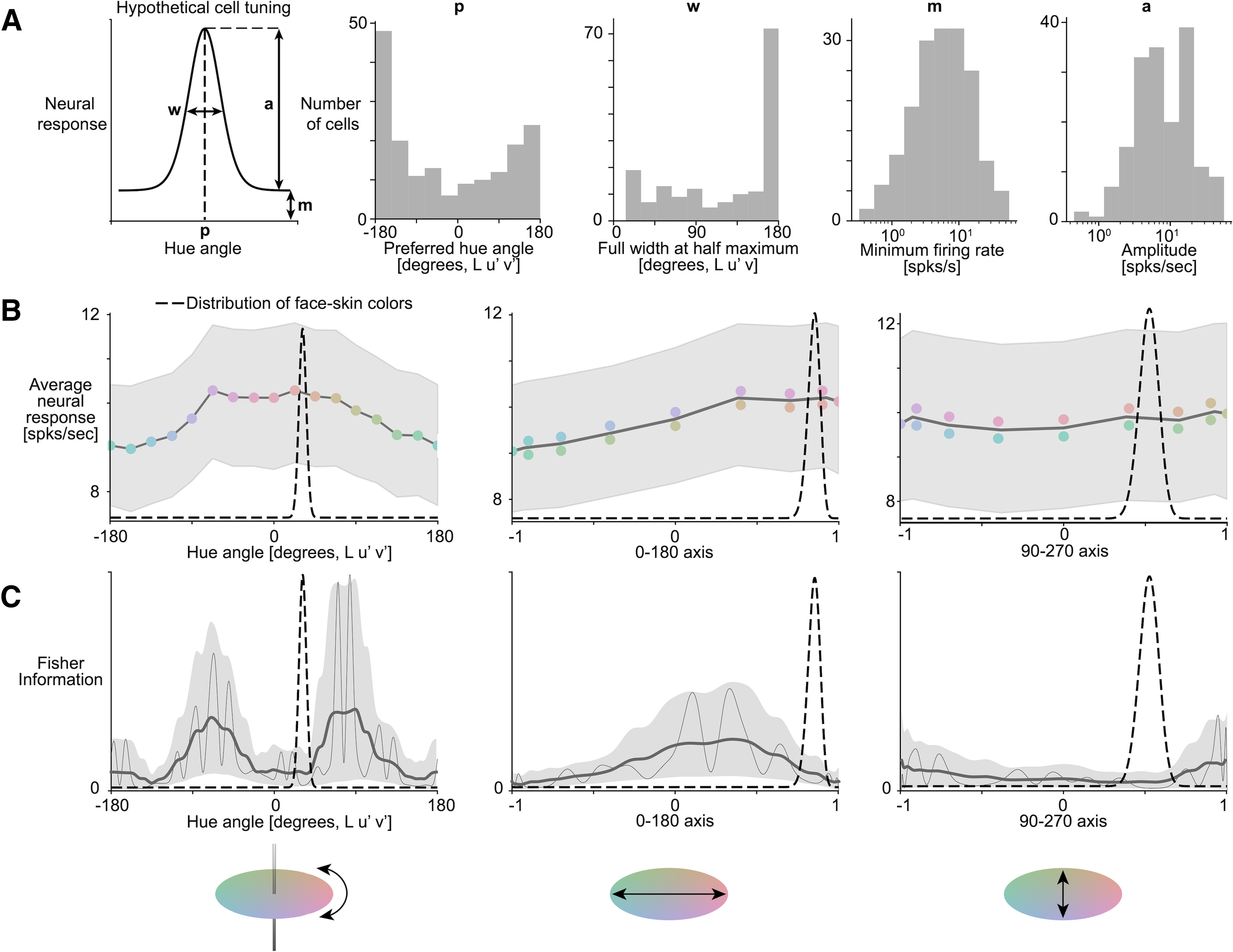
Analysis of the Information represented in the population. ***A***, Parameters of the von Mises fits over the 173 face-selective cells used to compute the population information. ***B***, Average net firing rate across the population. ***C***, Population Fisher information (thin line), smoothed Fisher information (thick line), and 95% confidence intervals. On both panels, the dashed line represents the distribution of natural face skin color, and the *y*-axis limits for Fisher information is kept constant across all three analyses. The left column corresponds to the analysis performed in CIELUV space, the middle one to the analysis projected along the greener to redder chromatic axis (positive values indicating redder), and the last one to the analysis projected along the bluer to yellower chromatic axis (positive values indicating yellower).

The data presented above quantify the color-tuning properties of face cells. If the color responses of face-selective cells reflect a role these cells play in discriminating face colors, we predicted that the Fisher information of the population would correspond to the distribution of face colors. The color statistics of face skin are available in a large database of calibrated measurements derived from multiple ethnicities (Xiao et al., 2017). We assume that the color statistics of bare macaque face skin shows a comparable bias to that found across humans (and we assume that neural measurements in macaque monkeys extend to the human case). Figure 11*C* shows the Fisher information computed for the neural data as a function of hue angle using von Mises function to describe the cells’ response (Fig. 11*A*). Superimposed on the panels is the distribution of face colors (dashed curves). The peak of the distribution of face colors does not correspond to maxima in the Fisher information; to the contrary, the likely colors of faces correspond to a dip in the Fisher information, which implies that the population is poor at discriminating the colors of faces. We did the same analysis projecting the original data along the 0–180° axis in CIELUV space, which approximates the L-M axis (Fig. 11*B*,*C*, middle panels), to evaluate whether face cells contain more information about the color component of faces that is most relevant for dynamic social signaling (the red pole of the green-red axis; Hasantash et al., 2019). In this analysis, the Fisher information peaks for reddish colors, consistent with the idea that face cells are color-tuned in a way that can contribute to the discrimination of L>M values. For comparison, Figure 11*B*,*C*, right panels, shows the analysis for data along the vertical axis in CIE color space, which approximates the S-cone axis. Face-selective cells did not show selective tuning along the S-cone axis; the Fisher information analysis implies that the cells do not carry as much information along the S-cone axis as they do along the L-M axis.

## Discussion

The population of face-selective neurons showed broad color tuning with a bias for reddish colors. The Fisher information, which reflects how well the neural population can discriminate among colors, shows a dip which coincides with the peak in the distribution of face colors measured across human ethnicities (Xiao et al., 2017). This pattern of results implies that face-selective cells in macaque IT are not optimally tuned to discriminate the colors of human faces. It is conceivable that face-selective neurons in macaque IT are optimally tuned to discriminate macaque face colors, although this would require a substantial difference in the colors of macaque faces compared with human faces, which is unlikely since the primary determinants of face coloring (oxygenated hemoglobin and melanin) are the same in both species. It is also conceivable that the color responses of the neurons may be stronger if color was manipulated in spatial patterns on the face to reflect the spatial distribution of natural color changes over the face. Thus, the color responses we report provide a lower bound. The color tuning curves were measured using images that preserve the normal luminance contrast relationships of face photographs. In a second series of experiments, we found that face-selective cells were not very responsive to pure color (equiluminant) images of faces, which underscores the importance of luminance contrast for face selectivity (Ohayon et al., 2012), and provides single-unit evidence supporting the fMRI observation that face patches respond more strongly to luminance contrast compared with equiluminant color (Lafer-Sousa and Conway, 2013). Taken together with prior work, the research supports the idea that color-specific information related to the discrimination of face colors is likely handled by neural circuits that are independent of face patches. This interpretation is consistent with the multistage parallel processing framework of IT, in which face-biased regions are largely non-overlapping with color-biased regions (Conway, 2018).

We related the neurophysiology results to behavior using an information framework. Optimal neural coding suggests that there should be a good match between neural tuning and the statistics of those parts of the environment that are relevant (Simoncelli and Olshausen, 2001; Ganguli and Simoncelli, 2010). Faces occupy a distinct gamut in color space (Crichton et al., 2012; Chauhan et al., 2015; Xiao et al., 2017). If face-selective cells participate in discriminating among the colors of faces, the Fisher information of the population of neural responses should correspond to the distribution of face colors. The neurophysiological results refute this prediction. Most of the significantly color-tuned face-selective neurons cells were best described as having broad tuning, with a single peak in the color-tuning function. On average, the color-tuning peaks across cells were to warm (L>M) colors (Fig. 7), corresponding to the typical colors of faces. The Fisher information curve is bimodal and the color-discrimination potential of face-selective neurons is therefore worse for face colors compared with greens and purples (Fig. 11, peaks at 84 and −66 hue angle).

These results suggest that some other population of neurons is responsible for discriminating the colors of faces. Functional MRI response patterns in both macaque monkeys and humans show a multistage organizational scheme governed by a repeated eccentricity template, in which color-biased tissue is sandwiched between face-selective tissue (foveal biased) and place-selective tissue (peripheral biased) in parallel streams along the length of the ventral visual pathway (Lafer-Sousa and Conway, 2013; Lafer-Sousa et al., 2016; Conway, 2018). This organization provides the possibility that color-specific information about objects, including faces, could be extracted by neural circuits besides the face patches. But we note that within the face-selective population we studied, some cells were color-tuned with peak tuning away from reddish colors; these cells were, curiously, the most color selective in the population (Fig. 6, top rows). These neurons may represent a distinct category of face-selective cells that could, conceivably, be optimally tuned to discriminate the colors of faces.

What role, if any, does the color tuning of face cells play in visual processing? We consider three possibilities. First, the information content with regards to color was not zero, so the cells could discriminate face colors, but non-optimally. Second, the color component of faces to which humans are most sensitive, regardless of race, is the aspect that varies in response to changes in emotion and health, which is encoded selectively along the L-M chromatic axis (Hasantash et al., 2019). Could the color tuning of face-selective neurons optimally discriminate just this component? Consistent with this possibility, we found that the average tuning and the Fisher information increased for stimuli with larger L>M values. The pattern of results is similar to the ramp-tuning functions of face-selective neurons for other stimulus features (Freiwald et al., 2009). Thus, the selectivity we observed implies that the extent of L-M color contrast in a face is a relevant feature encoded by face-selective neurons. Finally, we wonder whether the color tuning may serve to enhance the face-discrimination computations of the neurons. On average, faces have a warmer coloring than backgrounds (Rosenthal et al., 2018). It is plausible that the color responses would increase the firing rates of face-selective neurons when a face is encountered. Such modulation would presumably promote the role of these neurons in face recognition. According to this interpretation, the modulation by color of face-selective cells is analogous to the modulation of neural activity manifest when a subject engages in an attentional task (Wurtz and Mohler, 1976; Treue, 2001; Maunsell, 2015).

**Figure 12.**
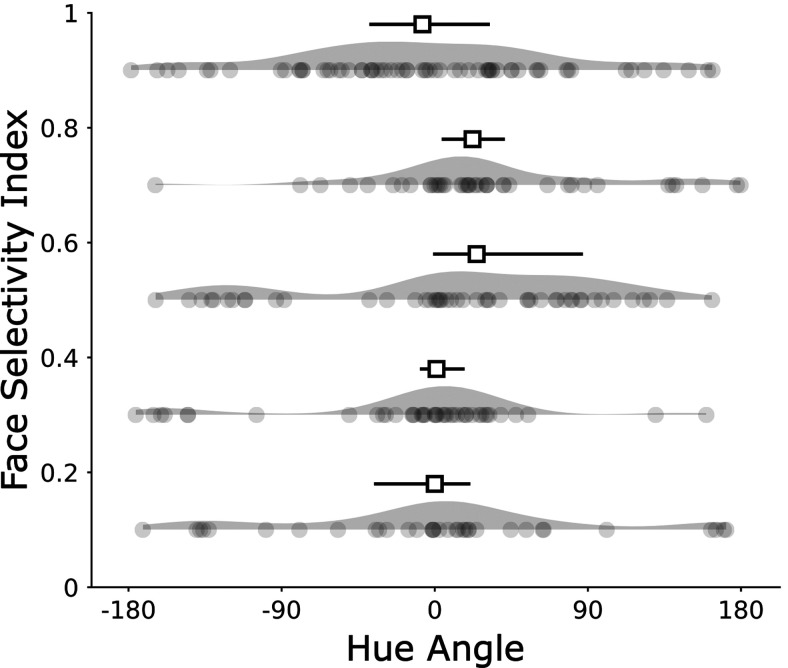
Hue preference by FSI. Each dot represents one cell in one bin of 0.2. Median and 95% confidence intervals on the median for each bin are represented above the kernel density estimate of the distribution. The mean FSI across all 234 cells is 0.55.

Is the color tuning we describe specific for faces? Quantitative analysis of the color statistics of those parts of scenes that we label, shows that objects tend to be systematically biased compared with backgrounds: objects, not just faces, tend to be distinguished from backgrounds along the u’ direction of color space, which corresponds, roughly, to warm coloring (Gibson et al., 2017; Conway et al., 2020). Tuning to warm coloring may therefore facilitate the computations of many cells in IT, not just face-selective neurons. This hypothesis is supported by fMRI maps of the color tuning across IT, which show a band running along the posterior-anterior axis that is more strongly modulated by the likely colors of objects (Conway, 2018; Rosenthal et al., 2018). This band is centered on the face patches but is not restricted to them. Consistent with the fMRI results, we found that the non-face-selective cells, often found on the margins or just outside of face patches, also showed a weak bias for warm colors (Fig. 12). Moreover, others have reported that the optimal stimuli for IT cells are often of warm coloring (Ponce et al., 2019). We speculate that the modulation by color is likely not a specific property of face cells but may reflect a feature of IT that facilitates the computation by IT of object recognition generally.

Although most cells had a single peak in the color-tuning function, some face-selective neurons were best fit by two peaks, with maximum power to the second harmonic in the Fourier analysis (see example cell #1; Fig. 4). The color selectivity of this subset of neurons, assessed as the phase angle of the second harmonic, was aligned with the S-cone axis in color space (Fig. 7). Is the tuning to the second harmonic meaningful? One possible concern could have been that these responses reflect a luminance artifact: estimates of equiluminance may not be accurate, especially for colors that modulate S-cones (Vos, 1978). We avoided this potential pitfall in the present work because the stimuli preserved the luminance contrast of the original images: the blackest and whitest regions in the colored versions of each image were the same as in the original image. Any luminance artifacts attributed to vagaries in the determination of equiluminance would be masked by preservation of the luminance contrast of the original image.

The response bias to colors along the S axis was surprising; we think the results provide the first measurements of color tuning biases within extrastriate cortex that reflect the cardinal mechanisms. The cardinal mechanisms correspond to the color tuning of the cone-opponent cells that represent the first postreceptoral stage of color encoding and are reflected in the anatomy and physiology of the lateral geniculate nucleus (Derrington et al., 1984; Martin et al., 2001; Sun et al., 2006; Roy et al., 2009). The cardinal mechanisms are evident in behavioral work that is thought to isolate these subcortical contributions to color vision (Krauskopf et al., 1982; Eskew, 2009). The observation that cortical cells reflect the cardinal mechanisms is surprising because the distinct chromatic signatures associated with the cardinal mechanisms diffuse near the input layers to primary visual cortex (Tailby et al., 2008), and the organization of color undergoes progressively more uniform representation of color space through the visual-processing hierarchy (Bohon et al., 2016; Liu et al., 2020). The present results show that chromatic signatures corresponding to the cardinal mechanisms reemerge in extrastriate cortical circuits far along the putative visual-processing hierarchy, and they raise the possibility that the behavioral results reflecting the cardinal mechanisms may derive from responses not only of subcortical circuits, but also of extrastriate circuits.

## Synthesis

Reviewing Editor: Morgan Barense, University of Toronto Decisions are customarily a result of the Reviewing Editor and the peer reviewers coming together and discussing their recommendations until a consensus is reached. When revisions are invited, a fact-based synthesis statement explaining their decision and outlining what is needed to prepare a revision will be listed below. The following reviewer(s) agreed to reveal their identity: Hidehiko Komatsu.

The reviewers and I agreed that this is an elegant study that provides a novel and important advance regarding colour representation in face-selective cells. Each reviewer raised specific suggestions and comments, which we believe will strengthen the manuscript. We look forward to receiving the revision, and I thank you for submitting to eNeuro.

REVIEWER 1:

General

In this study, the authors studied neural representation of color in face patches (ML and AL) of the macaque monkeys that were identified by fMRI. We are very sensitive to the change in visual features on faces including color, and the question addressed in the present study is an interesting one. In addition, cerebral achromatopsia often co-occur with prosopagnosia, and fMRI studies have shown that face patch system and color patch system are adjacently located. These observation also raise question as to whether and how there is interaction between face selectivity and color selectivity at the neuron level. It is expected this study also give some answers to such a question.

The authors recorded neuron activities from fMRI- identified face patches, and tested neural responses to achromatic images of various objects including human and monkey faces. Color selectivity was tested by presenting luminance matched face images having various hues defined on CIE Luv chromaticity diagram that have the same saturation. The authors found that a minority of face selective neurons (44/173) were significantly modulated by color, although luminance contrast seems the main determinant of the responses. There is clear bias in color tuning to warm colors that appears to coincide with the distribution of face colors. However, analysis employing Fisher information suggests that color selectivity of face-selective neurons is not optimally tuned to discriminate face colors.

Experiments are conducted carefully in general. This study gives important new information on the color-related properties of neurons in the face patch, and help to understand the relationship between face processing and color processing in the inferior temporal (IT) cortex. I have some comments on the results and analysis as listed below.

Specific comments:

Major

1. Fisher information depends on the distribution of tuning peaks, tuning widths, and amplitude modulation across the neural population. Figure 11 shows the results of the computation of Fisher information and its relation to the average neural responses and the distribution of face-skin colors. To evaluate this analysis, it is necessary to see the distribution of the parameters relevant to Fisher information, namely tuning peaks, tuning widths and amplitude. So these data should be provided as a figure.

2. An issue related to the above point is that in this study, color tuning is modelled with cosine wave (p. 12, last paragraph). If so, tuning width is constant, and validity of using Fisher information is not clear. I wonder whether the authors compared fitting with cosine function and von Mises tuning. In addition, why did the authors use exponential distribution to model the tuning in the cone contrast space?

3. One interesting result of this study is that face selective neurons are mainly activated by luminance contrast of the face image. However, it should be noted that there is no unique scale to compare different axes in the color space, such as L-M axis, S axis and achromatic axis. Especially, constant saturation stimuli such as those used in this study can employ only relatively low color contrast stimuli compared with the entire gamut of the chromaticity diagram. If vivid color stimuli are used, response modulation may become much larger. The point is that as long as the color stimuli with constant saturation (or constant cone contrast) is used, the conclusion about the relative contribution between color and luminance signal should be tentative. Experiment using isoluminant images provide important information with this regard, but this is only one way to look at the interaction between color and luminance signals.

4. In the analysis shown in the right column of Fig. 12A, neural response to each hue was projected on the L-M axis. Procedure of this analysis is not clear, and should be described in more detail. I guess, coordinate on L-M axis after projecting each color is projected on this axis is used for the analysis. If this is correct, I wonder why L-M axis is used for this analysis. I think the same analysis should be made for each direction in the chromaticity diagram before making conclusion on which axis can best describe the neural tuning. In addition, for such an analysis, I think CIE Luv space should be used instead of DKL space. I will describe on this last point in my next comment.

5. In many places in this paper, neural responses are associated with L-M axis of DKL space. This can be done in Discussion, but it is not appropriate to do this in describing the results. The stimuli used in the experiments are defined on CIE Luv space, one of uniform color space, so the results should be described in relation to this space. One problem of describing the results with respect to Luv diagram may be that there is no clear physiological meaning for major axes (e.g. 0deg, 90deg, 180deg, 270deg), but it is also problematic to relate the results with cardinal axes of DKL space that has clear association only with subcortical color processes. For example, on line 4 from the bottom of page 4, it is stated that “the data points to the right of the plot converge on 0 degrees (the L>M pole of the L-M axis)”. ‘L>M pole of the L-M axis’ should be rephrased as ‘red pole’ to exclude contamination of assumption on physiological mechanism. Furthermore, Fig. 7 A, B shows that distribution of color tuning is aligned on 0deg in Luv diagram, but it is deviated from L-M axis.

6. The results of this study shows there is clear bias of color tuning to warm colors. As the authors mentioned in the text, previous study by the same group has shown that object colors tend to have warm colors and neural activities in IT are strongly modulated by the object colors (Rosenthal et al. 2018). Those observations naturally raise a question that is whether the color tuning of neurons in face patch is the same or different from those outside the face patch. Argument on this point will help readers to understand the color representation in IT.

Minor

1. Abstract and Significance: It is not clear what ‘dynamic L-M component’ or ‘dynamic aspects of face coloring’ mean. Some explanation should be given.

2. Page 5, line 9: ‘constant gray value but different saturation’: ‘gray value’ should be ‘hue’.

3. Figure 5 legend: It is stated that red color represents significantly color tuned cells and black represents ‘the population’. Does the ‘population’ for black color include all 173 neurons, or 129 neurons that exclude color tuned neurons? This point should be made explicit.

4. Page 3, last line: ‘Most cells (71/173)’ I think ‘most’ is not appropriate word for this ratio.

5. Page 4, second paragraph: “Figure 3 and 4 provide evidence that some face-selective cells were sensitive to color.” Figure 3 illustrates visual stimuli used for the experiments, so only Figure 4 should be mentioned.

6. Figure 10A: This figure shows the average above-background level firing rate of neural population. The peak of distribution is at about 292.5 deg, not 0 deg. This seems to disagree with the distribution of color tuning shown in Figures 7 and 8 that are clearly at 0 deg. Some explanation on this disagreement should be given. It may be also useful to show the distribution of tuning peaks, tuning widths, and amplitude modulation across the neural population as I mentioned in my major comment #1 above.

REVIEWER 2:

This manuscript focuses on color responses in face patches of macaque inferior temporal cortex. What does the role of color play in neural representation of faces? To study this question, the authors use the technique of fMRI guided microelectrode recording to record color responses of IT middle and anterior face patches. IT is the high-stage cortex in the primate ventral visual pathway, and it play a vital role in color and object perception. The signals of visual features are commonly processed in modular manner along the visual hierarchy, however, what are neural mechanisms underlying combined visual features in a feature-specific module is much less studies. As the authors noted, “many studies of face perception use exclusively colorless images", there lacks a systemic study of how color signals are represented in face-selective patches. To my knowledge this work is the first quantitative study of color-tuning property of face-selective neurons in alert primate. This is an outstanding work in terms of scientific question the authors address and the high-quality data they obtained.

This study confirmed the face-selective neuron do carry color signals, and most of them have broad color tuning. The cortical response was previously found to be biased to endspectral colors (red and blue) in primary visual cortex (Tootell et al., 1988; Garg et al., 2019), which mainly reflects the contrast along the L-M axis (Valverde et al, 2012). A recent study found this endspectral bias diminishes progressively from V1 to V2, then to V4. In V4, the response bias to blue is disappeared and there only remains some bias to warm colors (Liu et al, 2020). This study finds that most of the color tuning in IT face patches bias towards the warm color (L>M pole of L-M axis), suggesting a continuous processing of warm color signal along the ventral hierarchy. More surprisingly, the authors found the modulation of S-cone axis in IT. Parvocellular (L-M) and koniocellular (S-(L+M)) signals are believed to converge in V1 and be integrated along the visual hierarchy, but the modulation of koniocellular signal can be hardly measured in previous studies. This finding of S-cone axis modulation may provide a novel view of understanding color processing mechanisms.

Minor comments:

1. Figure 5: What is the meaning of the solid lines? I assume it may indicate the average normalized amplitude across all the face-selective neurons in ML and AL. This information is not mentioned in the figure legend.

2. Figure 6: What is the meaning of the last sentence of figure legend “a maximal preference for one hue would be indicated by black for that hue and white for all others”? I think it is conflicting with the gray bar of cell response.

3. Figure 7A: Which harmonic component the left panel and right panel correspond to are not described clearly in the legend.

4. Figure 12: The distribution of gray dots has demonstrated that the color tuning of non-face-selective cell bias to warm colors, but the statistical presentation using median plus 95% confidence interval seems not to be the optimal way to present this conclusion. Can the authors present this result in another manner, such as histogram distribution?

## Author Response

We thank the reviewing editor and the reviewers for their constructive feedback. Point-by-point responses

are provided below.

Manuscript Instructions

- Please re-organize your manuscript so the Materials and Methods section follows immediately after the

Introduction section.

Done.

Synthesis of Reviews:

Computational Neuroscience Model Code Accessibility Comments for Author (Required):

N/A

Significance Statement Comments for Author (Required):

In addition to addressing the specific comments provided by Reviewer 1, I felt that this statement could be

edited to make it more accessible to a non-specialist audience. As it currently reads, the significant

advance may not be apparent to all.

We have updated the Significance Statement to make it more accessible to a non-specialist audience.

Comments on the Visual Abstract for Author (Required):

N/A

Synthesis Statement for Author (Required):

The reviewers and I agreed that this is an elegant study that provides a novel and important advance

regarding colour representation in face-selective cells. Each reviewer raised specific suggestions and

comments, which we believe will strengthen the manuscript. We look forward to receiving the revision,

and I thank you for submitting to eNeuro.

Thank you for the nice feedback.

REVIEWER 1:

General

In this study, the authors studied neural representation of color in face patches (ML and AL) of the

macaque monkeys that were identified by fMRI. We are very sensitive to the change in visual features on

faces including color, and the question addressed in the present study is an interesting one. In addition,

cerebral achromatopsia often co-occur with prosopagnosia, and fMRI studies have shown that face patch

system and color patch system are adjacently located. These observations also raise question as to

whether and how there is interaction between face selectivity and color selectivity at the neuron level. It is

expected this study also give some answers to such a question.

The authors recorded neuron activities from fMRI- identified face patches, and tested neural responses to

achromatic images of various objects including human and monkey faces. Color selectivity was tested by

presenting luminance matched face images having various hues defined on CIE Luv chromaticity diagram

that have the same saturation. The authors found that a minority of face selective neurons (44/173) were

significantly modulated by color, although luminance contrast seems the main determinant of the

responses. There is clear bias in color tuning to warm colors that appears to coincide with the distribution

of face colors. However, analysis employing Fisher information suggests that color selectivity of faceselective neurons is not optimally tuned to discriminate face colors.

Experiments are conducted carefully in general. This study gives important new information on the colorrelated properties of neurons in the face patch, and help to understand the relationship between face

2

processing and color processing in the inferior temporal (IT) cortex. I have some comments on the results

and analysis as listed below.

Specific comments:

Major

1. Fisher information depends on the distribution of tuning peaks, tuning widths, and amplitude

modulation across the neural population. Figure 11 shows the results of the computation of Fisher

information and its relation to the average neural responses and the distribution of face-skin colors. To

evaluate this analysis, it is necessary to see the distribution of the parameters relevant to Fisher

information, namely tuning peaks, tuning widths and amplitude. So these data should be provided as a

figure.

We have now fitted the cell’s response with von Mises function and present the distribution of parameters

in the new version of Figure 11, panel A (see also response to 4.)

2. An issue related to the above point is that in this study, color tuning is modelled with cosine wave (p.

12, last paragraph). If so, tuning width is constant, and validity of using Fisher information is not clear. I

wonder whether the authors compared fitting with cosine function and von Mises tuning. In addition, why

did the authors use exponential distribution to model the tuning in the cone contrast space?

We initially used a cosine model to remain coherent with the Fourier analysis that showed a higher

amplitude in the first harmonic component. However, we see the rationale behind using von Mises fits

that allow variation in tuning widths, is more commonly used in analyses of circular variables, and is used

by ourselves in Figure 1 to illustrate putative population information.

Using an exponential distribution to model the tuning in the cone contrast space was purely pragmatic.

We looked at the response along that space and compared several models, exponential was a the best

one. We were not particularly happy about that process, and have now removed that analysis, projecting

directly onto the CieLUV chromatic axis space as suggested, and straightforwardly projected the same

circular von Mises model onto the axis. (see also response to 4.)

3. One interesting result of this study is that face selective neurons are mainly activated by luminance

contrast of the face image. However, it should be noted that there is no unique scale to compare different

axes in the color space, such as L-M axis, S axis and achromatic axis. Especially, constant saturation

stimuli such as those used in this study can employ only relatively low color contrast stimuli compared

with the entire gamut of the chromaticity diagram. If vivid color stimuli are used, response modulation may

become much larger. The point is that as long as the color stimuli with constant saturation (or constant

cone contrast) is used, the conclusion about the relative contribution between color and luminance signal

should be tentative. Experiment using isoluminant images provide important information with this regard,

but this is only one way to look at the interaction between color and luminance signals.

We have added to the text a discussion of this important issue. As the reviewer points out,

because there is no accepted metric for relating color contrast and luminance contrast (Shevell &

Kingdom, 2008), it is often difficult to compare responses to equiluminant stimuli with responses to

luminance contrast stimuli. In the present study, this difficulty is mitigated for several reasons. First, the

maximum color contrast of the equiluminant stimuli was the highest that the gamut of the display could

produce. If color were a sufficient drive of the neural activity of face-selective neurons, the equiluminant

stimuli we used should elicit strong responses. Second, using stimuli of comparable color and luminance

contrast, other neurons in the visual system show clear preferences for the color stimuli (in V1: Conway,

2001, Horwitz and Hass, 2012, Johnson et al, 2004; in V4, Conway et al, 2007, Bohon et al, 2016; in IT,

Komatsu 1998; Lafer-Sousa and Conway, 2013), confirming that the stimuli are capable of eliciting strong

responses when neurons are responsive to color.

4. In the analysis shown in the right column of Fig. 12A, neural response to each hue was projected on

3

the L-M axis. Procedure of this analysis is not clear, and should be described in more detail. I guess,

coordinate on L-M axis after projecting each color is projected on this axis is used for the analysis. If this

is correct, I wonder why L-M axis is used for this analysis. I think the same analysis should be made for

each direction in the chromaticity diagram before making conclusion on which axis can best describe the

neural tuning. In addition, for such an analysis, I think CIE Luv space should be used instead of DKL

space. I will describe on this last point in my next comment.

We thank the reviewer for the comments related to the population information analysis (Figure 11). We

now present the population information analysis using von Mises fits, along the CieLUV circular space,

and along the 180-0 (green-red) and the 270-90 (blue-yellow) chromatic axes.

Comparing von Mises to cosine fits actually showed that von Mises provide descriptively a better fit than

cosine (MdMSE_vonMises = 0.38 spks/s, MdMSE_cosine = 0.46 spks/s), but not significantly better (Mann-Whitney

test, p=.18). Overall, results using these fits and subsequent conclusions are very similar to the ones

obtained using cosine fits, i.e 1. along the CieLUV space, peaks in population in the yellow and blue,

don’t align with the distribution of face color, 2. We observe higher modulation in firing rate and population

information along the red-green axis than along the blue-yellow axis.

5. In many places in this paper, neural responses are associated with L-M axis of DKL space. This can be

done in Discussion, but it is not appropriate to do this in describing the results. The stimuli used in the

experiments are defined on CIE Luv space, one of uniform color space, so the results should be

described in relation to this space. One problem of describing the results with respect to Luv diagram may

be that there is no clear physiological meaning for major axes (e.g. 0deg, 90deg, 180deg, 270deg), but it

is also problematic to relate the results with cardinal axes of DKL space that has clear association only

with subcortical color processes. For example, on line 4 from the bottom of page 4, it is stated that “the

data points to the right of the plot converge on 0 degrees (the L>M pole of the L-M axis)”. ‘L>M pole of the

L-M axis’ should be rephrased as ‘red pole’ to exclude contamination of assumption on physiological

mechanism. Furthermore, Fig. 7 A, B shows that distribution of color tuning is aligned on 0deg in Luv

diagram, but it is deviated from L-M axis.

We have explicitly stated in the manuscript that the stimuli were obtained using colors defined by CIE,

and the justification for this decision. Colors can be linearly transformed from one space to another using

straightforward algorithms. Because the DKL space relates to physiological coordinates, we find it useful

to identify which of the colors defined in CIE correspond to the poles of the cone-opponent axes of the

DKL color space. We think that having this information in the figures and the results is helpful and valid.

We state this at first mention of DKL in the manuscript (accompanying Figure 2).

6. The results of this study show there is clear bias of color tuning to warm colors. As the authors

mentioned in the text, previous study by the same group has shown that object colors tend to have warm

colors and neural activities in IT are strongly modulated by the object colors (Rosenthal et al. 2018).

Those observations naturally raise a question that is whether the color tuning of neurons in face patch is

the same or different from those outside the face patch. Argument on this point will help readers to

understand the color representation in IT.

The reviewer raises an important point, which we have underscored by doing the analysis shown in

Figure 12. That figure shows the color tuning of non-face-selective neurons located near face patches;

these cells also showed a bias for warm colors, that was not distinguishable from the bias we found for

face-selective neurons. This is consistent with prior work (Rosenthal et al., 2018), and suggests that the

warm bias of face-selective neurons is not face specific. We discuss these issues in the discussion: Is the

color tuning we describe specific for faces? Quantitative analysis of the color statistics of those parts of

scenes that we label, shows that objects tend to be systematically biased compared to backgrounds:

objects, not just faces, tend to be distinguished from backgrounds along the u’ direction of color space,

which corresponds, roughly, to warm coloring (Gibson et al., 2017; Conway et al., 2019). Tuning to warm

coloring may therefore facilitate the computations of many cells in IT, not just face-selective neurons. This

hypothesis is supported by fMRI maps of the color tuning across IT, which show a band running along the

posterior-anterior axis that is more strongly modulated by the likely colors of objects (Conway, 2018;

4

Rosenthal et al., 2018). This band is centered on the face patches but is not restricted to them.

Consistent with the fMRI results, we found that the non-face-selective cells, often found on the margins or

just outside of face patches, also showed a weak bias for warm colors (Figure 12). Moreover, others

have reported that the optimal stimuli for IT cells are often of warm coloring (Ponce et al., 2019). We

speculate that the modulation by color is likely not a specific property of face cells but may reflect a

feature of IT that facilitates the computation by IT of object recognition generally.

Minors

1. Abstract and Significance: It is not clear what ‘dynamic L-M component’ or ‘dynamic aspects of face

coloring’ mean. Some explanation should be given.

We have replaced this language in the significance statement.

2. Page 5, line 9: ‘constant gray value but different saturation’: ‘gray value’ should be ‘hue’.

This has been corrected.

3. Figure 5 legend: It is stated that red color represents significantly color tuned cells and black

represents ‘the population’. Does the ‘population’ for black color include all 173 neurons, or 129 neurons

that exclude color tuned neurons? This point should be made explicit.

We have now added that the number of cells. We chose to show the whole population of 173 neurons,

and not only the non-significant ones to preserve coherence with the color tuning analyses, that present

data over the entire population.

4. Page 3, last line: ‘Most cells (71/173)’ I think ‘most’ is not appropriate word for this ratio.

This has been corrected, replacing “most” by “many”.

5. Page 4, second paragraph: “Figure 3 and 4 provide evidence that some face-selective cells were

sensitive to color.” Figure 3 illustrates visual stimuli used for the experiments, so only Figure 4 should be

mentioned.

We have removed reference to Figure 3.

6. Figure 10A: This figure shows the average above-background level firing rate of neural population. The

peak of distribution is at about 292.5 deg, not 0 deg. This seems to disagree with the distribution of color

tuning shown in Figures 7 and 8 that are clearly at 0 deg. Some explanation on this disagreement should

be given. It may be also useful to show the distribution of tuning peaks, tuning widths, and amplitude

modulation across the neural population as I mentioned in my major comment #1 above.

Indeed, in Figure 10A we show the average above-background firing rate of the neural population. You

might have noted that the window used to compute that average is longer than the one used for the

detailed analyses of the color responses of the cells (we used here the full 400ms of 200ms of stimulus

and 200ms of blank between 2 stimuli). That was done in order to make neural and Bold responses more

comparable. However, the choice of window doesn’t change the pattern of results, and computing the

average firing rate above background over the windows used for the color tuning analyses (tailored for

each cell) yields a similar pattern with a peak at 292.5 deg (a second one at 45 deg).

We have shown in the analyses, and as you noted in Figure 7 and 8, that the overall population contains

energy in the first Fourier component and that the median peak of that component is 0 (or for the whole

population ∼13 degrees). However, we also show that the population has some energy in the second

component that peaks at ∼100 degrees. If only the first component was present, we would expect a peak

in the average population activity around 13, but if we take into account the second component, we would

counterintuitively expect a peak away from the peak of the first component and closer to the peak of the

second one. That point is illustrated left panel of the figure below. If we sum two cosine waves of similar

amplitude (the first component with a peak at 0, and the second one with a peak at 90), the sum presents

two peaks, at 76 and 283 deg. As the relative amplitude decreases between the two components, the

peak flattens and is pushed closer to the peak of the first component if its amplitude is higher. In the right

5

panel we illustrated the case corresponding to the population data, (peaks respectively at 13 and 100 deg

for the two components), if the second one has an amplitude of half the first one (closer to our dataset),

we would predict 2 peaks at 80 and 310. We attribute deviances from that to aliasing and the contribution

of higher frequency components.

We have added one sentence to the legend of figure 10 to hint averted readers towards that explanation.

REVIEWER 2:

This manuscript focuses on color responses in face patches of macaque inferior temporal cortex. What

does the role of color play in neural representation of faces? To study this question, the authors use the

technique of fMRI guided microelectrode recording to record color responses of IT middle and anterior

face patches. IT is the high-stage cortex in the primate ventral visual pathway, and it play a vital role in

color and object perception. The signals of visual features are commonly processed in modular manner

along the visual hierarchy, however, what are neural mechanisms underlying combined visual features in

a feature-specific module is much less studies. As the authors noted, “many studies of face perception

use exclusively colorless images", there lacks a systemic study of how color signals are represented in

face-selective patches. To my knowledge this work is the first quantitative study of color-tuning property of

face-selective neurons in alert primate. This is an outstanding work in terms of scientific question the

authors address and the high-quality data they obtained.

This study confirmed the face-selective neuron do carry color signals, and most of them have broad color

tuning. The cortical response was previously found to be biased to endspectral colors (red and blue) in

primary visual cortex (Tootell et al., 1988; Garg et al., 2019), which mainly reflects the contrast along the

L-M axis (Valverde et al, 2012). A recent study found this endspectral bias diminishes progressively from

V1 to V2, then to V4. In V4, the response bias to blue is disappeared and there only remains some bias to

warm colors (Liu et al, 2020). This study finds that most of the color tuning in IT face patches bias towards

the warm color (L>M pole of L-M axis), suggesting a continuous processing of warm color signal along

the ventral hierarchy. More surprisingly, the authors found the modulation of S-cone axis in IT.

Parvocellular (L-M) and koniocellular (S-(L+M)) signals are believed to converge in V1 and be integrated

along the visual hierarchy, but the modulation of koniocellular signal can be hardly measured in previous

studies. This finding of S-cone axis modulation may provide a novel view of understanding color

processing mechanisms.

We thank the reviewer for their careful and constructive review. As the reviewer points out, there is a long

history of work documenting color responses of cells at many stages of the visual pathway. We have

updated the end of the discussion in an attempt to reflect this rich history, including a reference to Liu et

al (2020), which provides an excellent recent summary of the transformation of color signals through the

visual-processing hierarchy from V1 to V4.

Minor comments:

1. Figure 5: What is the meaning of the solid lines? I assume it may indicate the average normalized

amplitude across all the face-selective neurons in ML and AL. This information is not mentioned in the

figure legend.

6

We have now added this information to the legend (the solid line indeed represented the average across

face patches).

2. Figure 6: What is the meaning of the last sentence of figure legend “a maximal preference for one hue

would be indicated by black for that hue and white for all others”? I think it is conflicting with the gray bar

of cell response.

We agree that the statement is unclear, we wanted to illustrate the color scale by mentioning a

hypothetical cell - narrowly tuned - that would fire more in response to only one hue and respond less -

but equally less to all other hues. In that case the color for that hue would be black and the one for all

others would be white. But after some more thoughts, this example - far from the observed responses -

does not help the reader understand the figure so we have now removed it.

3. Figure 7A: Which harmonic component the left panel and right panel correspond to are not described

clearly in the legend.

We have now added this information to the legend for both left and right panel.

4. Figure 12: The distribution of gray dots has demonstrated that the color tuning of non-face-selective

cell bias to warm colors, but the statistical presentation using median plus 95% confidence interval seems

not to be the optimal way to present this conclusion. Can the authors present this result in another

manner, such as histogram distribution?

In Figure 12 we want to illustrate 2 points: first, indeed, that non face-selective cells also show a bias for

warm colors, and second, that the warm bias doesn’t seem to differ for different strength of faceselectivity for cells that are face-selective. We agree that the Figure as it was, was hard to read because

the data points (transparent grey dots) are overlapping making it difficult to extract a representation of the

distribution, and even more difficult with the median and 95% confidence intervals overlayed. We have

now replotted the data with split-violins (allowing an easier visualization of the distributions of hue angles),

while still keeping the data points. Medians and 95% CI are now offset from the distribution making them

more readable. We believe that this new representation greatly improves the Figure.
